# Supply chain network rewiring dynamics at the firm level

**DOI:** 10.1093/pnasnexus/pgag091

**Published:** 2026-04-16

**Authors:** Tobias Reisch, András Borsos, Stefan Thurner

**Affiliations:** Complexity Science Hub Vienna, Vienna A-1080, Austria; Complexity Science Hub Vienna, Vienna A-1080, Austria; Department of Financial Systems Analysis, Central Bank of Hungary, Budapest 1013, Hungary; Complexity Science Hub Vienna, Vienna A-1080, Austria; Section for Science of Complex Systems, CeMDAS, Medical University of Vienna, Vienna A-1090, Austria; Santa Fe Institute, Santa Fe, NM 85701, USA; Supply Chain Intelligence Institute Austria, Vienna A-1080, Austria

**Keywords:** network evolution, supply chain networks, systemic risk, temporal networks, generative model

## Abstract

Supply chain networks (SCN) form the structural backbone of any society. They constitute the societal metabolism that literally produces everything for everybody by coordinating practically every single person on the planet. SCNs are by no means static but undergo permanent change through the entry and exit of firms and the rearrangement of supply relations. Here, we use a unique dataset to explore the temporal evolution of firms and their supplier–buyer relations of a national SCN. Monthly reported value added tax data from Hungary from 2014 to 2022 allows us to reconstruct the entire economy with 711,248 companies and 38,644,400 connections, covering practically every restructuring event of an entire economy at firm-level resolution. We find that per year about 25% of firms exit the SCN while 28% new ones enter. On average, 55% of all supply-links present in 1 year will not be present in the next. We report the half-life time of supply-links to be 13 months. New links attach super-preferentially to firms with a probability, $p(i)\propto k_{i}^{1.08}$, with $k_{i}$ firm *i*’s number of supply connections. We calibrate a simple statistical network generation model that reproduces the stylized characteristics of the dominant Hungarian SCN. The model not only reproduces local network features such as in- and out-degree distributions, assortativity, and clustering structure but also captures realistic systemic risk profiles. We discuss the present model in how rewiring dynamics of the economy is essential for quantifying its resilience and to estimate shock propagation.

Significance statement The way economies function largely depends on their underlying supply chain networks. Using a unique temporal dataset of firm-to-firm relations of a national supply chain, we describe the statistical properties of the entries and exits of firms and the relinking dynamics of the buyer–supplier links. We find that the network is by no means static but reconfigures at remarkable rates, leading to known emergent properties, such as efficiency and resilience. With this statistical information, we calibrate a large scale, generative supply chain network model to see if one can understand emergent economic properties from networks in a permanent state of rewiring. We correctly predict the emergent phenomenon of economic systemic risk and reproduce a number of network characteristics of the empirical supply chain.

## Introduction

The economy, ie the invention, production, distribution, consumption, usage, management, infrastructure, recycling, and disposing of almost all intermediate and final goods and services is organized through firms. At the firm level, most decisions in the economy are taken, for example what and how to produce their goods or services, who to hire, when to invest, how to innovate, and how to do administration, etc. Firms are connected to each other through buyer–supplier relations. Firms, together with the material and financial flows on the buyer–supplier links, form the backbone of every society’s metabolism—literally. They manage essential information of the flows of goods, products, production, investments, ideas, services, payments, etc. These relations not only produce all goods, services, food, buildings, and infrastructure, they also organize, educate, and maintain talents and workers, investments, etc.

**Figure 1:**
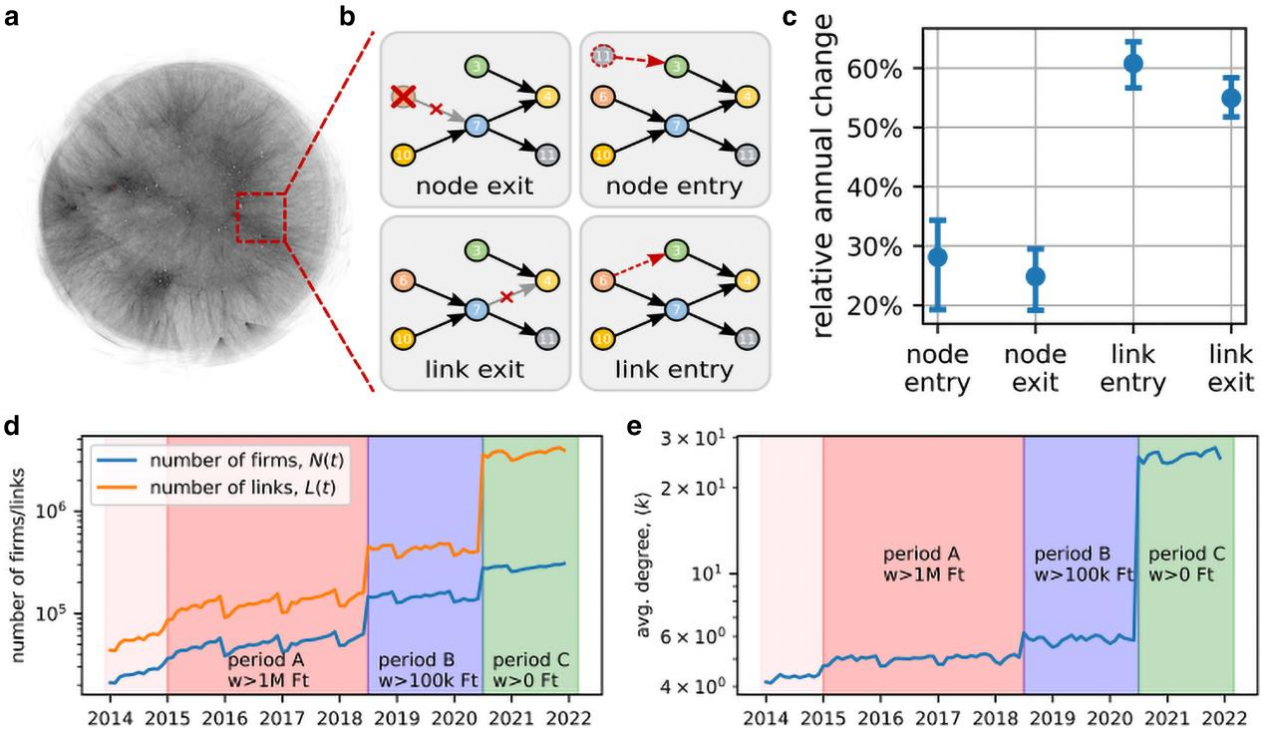
Temporal evolution of the Hungarian production network. a) Snapshot of the Hungarian SCN in 2017. Nodes represent 91,595 companies connected by about 230,388 supply relations. Layout: force-and-spring. b) Schematic view of the four elementary network reconfiguration steps. c) Relative annual rates of the network restructuring processes. Symbols show the average rate of change with respect to the previous year, bars denote the minimum and maximum values across the years 2015–2022, where we exclude the years 2018 and 2020, because there were changes in the reporting standards that distort the rates. d) Monthly number of active firms and links. The shading highlights periods with different reporting thresholds for the links. e) Average degree for each month. Same shading as (d). Note that despite the massive numbers of nodes and links entering and exiting, the network size and link density remain stable within periods with no change in reporting threshold. Panel a adapted from (10), with permission from the copyright holder.

The set of all buyer–supplier relationships within an economy are often referred to as *supply chains*, which is to some extent misleading, since most production processes (or sequences of labour steps) are not structured as simple linear chains, but these “chains” intersect, and constantly change over time to form complex structures that we call *supply chain networks* (SCN). For a depiction of a temporal snapshot of a national SCN, see Fig. 1a. SCN are central for understanding many economic processes such as innovation (1, 2), growth (3), development (4), greenhouse gas emissions (5–7), economic shock spreading (8–10), and resilience (11–13). Surprisingly, little is known about the economy at this “atomistic” scale of SCNs, in particular the structures, patterns, and laws behind the dynamical rewiring of the associated networks are hitherto unknown.

Economies are comprised of several hundred thousands to millions of companies that are connected through hundreds of millions to billions of buyer–supplier dependencies. Globally, there are an estimated 300 million firms with an estimated 13 billion supply links (14). Until recently, it seemed unimaginable to investigate the SCNs of entire economies at the firm level. The standard today is to look at an aggregation of companies into so-called industry sectors, such as the NACE industry classification scheme (15). First attempts to map a national economy on the industry level date back to Leontief’s work almost a century ago (16), which lead to the framework of *input–output economics* that still today is a workhorse for applied economics. Only recently empirical descriptions of SCNs on the firm-level arrived. The first studies, based on a large commercial dataset from Japan (17–19), report structural network features such as a scale free degree distribution, disassortativity, and sublinear scaling of sales with the number of supply connections. An early description of a European national SCN uses value added tax (VAT) data of Belgium to understand the individual firms’ network distance to final demand and their relationship with international trade (20). In recent years, several large datasets, many based on VAT data, were accumulated, including Hungary (10, 21), Ecuador (22), or Uganda (23); for a recent review, see Ref. (22). In countries where such data are not collected, SCNs have been reconstructed from alternative data sources, such as statistical surveys (24, 25), monetary transactions (26), or interfirm communication networks (27). For a review of the dramatic growth of datasets in recent years in terms of firms involved, see Ref. (14).

Most studies consider the SCNs as directed, weighted networks (17–22), while only some recognize their multilayer nature (10, 21, 24–26) that associates different products, services, or industries with layers of a multilayer network. The multilayer structure of SCNs is used as a key ingredient for reconstruction tasks from alternative data sources (24, 26, 27).

To understand the economy at the atomistic level, knowledge of national SCN structures alone is insufficient; one must also account for firms’ *production functions*, ie how inputs are transformed into outputs. Production functions vary: in a Leontief type, output is limited by the scarcest essential input (10); in a linear type, output is a linear combination of inputs and does not collapse when one input is missing. A more general functional form combining both was introduced in Ref. (10) and will be used here. Another widely applied form is the Cobb–Douglas production function, a multiplicative power-law (28).

An essential observation is that when combining the SCN topology with the production functions of the constituent firms the network is turned into a *hypergraph*, a generalized network structure that connects sets of nodes (inputs) with other sets (outputs) (29). The hypergraph structure is especially important for understanding the operation of SCNs on the systemic level, including shock propagation in production networks. It is essential for the appropriate quantification of *economic systemic risk* (10). In the following, we use the term “SCN” and keep its hypergraph structure in mind.

The final step for an atomistic understanding of the economy is to understand the dynamics of its SCNs and production functions. SCNs are subject to continuous restructuring, or rewiring. In Fig. 1b, we show the four elementary processes that take place when a SCN evolves in time. Firms can exit and enter the production network at certain rates. Whenever a firm exits all of its buyer–supplier connections (in- and out-links) vanish; if a new firm enters it establishes new links to existing firms. Buyer–supplier links typically are constantly updated between firms (link exit and link entry) both, in terms of strength (amount of goods/services exchanged), and who trades with whom. The underlying attachment mechanisms behind these highly dynamical link-updates are typically nontrivial and include details like firm strategy and technological decisions (30), price differences (31), geographic proximity of firms (32), current network structure (33, 34), personal preferences, and taste of decision makers and are therefore hard to quantify and might—at the current state—only be accessible on statistical grounds.

Here, we explore the temporal evolution of an empirical SCN on statistical grounds, by examining every entry and every exit of firms as well as the formation and termination of every buyer–supplier connection during almost a decade of the economic history of Hungary from 2014 to 2022, see Materials and methods. To this end, we use monthly reported VAT data in Hungary containing 711,248 companies and 38,644,400 connections. We cover practically every restructuring event of an economy at the firm-level resolution. We quantify monthly entry and exit rates for both firms and links and study the local conditions in the SCN under which link changes occur, in particular the degree of nodes that enter. For links that are generated between firms, we estimate the role of both the industry sector, and the size of customers and suppliers. Building on these results, we then develop a simple network generative model to understand the network properties that emerge from the microscopic restructuring processes. We include various aspects of previous network generative models, including (35–37) and choose a sparse parametrization that is exclusively based on microdata. The model covers firm entry and exit, spontaneous link generation and exit, as well as nontrivial attachment processes for buyer–supplier connections, see Results section. We then compare the structural network characterisitcs of the model with the observed empirical data, such as various degree distributions, assortativity structure, and local clustering. Finally, we test whether the model is able to realistically capture systemic risk and compare model systemic risk profiles with empirical ones. The employed systemic risk measure is the economic systemic risk index (ESRI) (10), a network centrality measure specifically designed to estimate the systemic risk created by every single firm in a SCN—up- and downward the SCN. ESRI takes production functions explicitly into account. Our model is data-driven in the sense that we measure *all* parameters directly from microdata—there are no free ad hoc chosen parameters remaining in the model.

There has been a series of previous attempts to model SCN formation under various aspects. To understand the in-degree distribution of the US production network, a generative network model was developed using firm-level microdata (38). Another early contribution (39) studies the decline of the New York garment industry and finds that preserving asymmetric (disassortative) links (in terms of degree) is important to retain the topology and functionality of the shrinking network. Subsequent studies (33, 34) emphasized the importance of the presently realized network structure for its evolution. Two theoretical studies show how input specific productivity and price differences can lead to the emergence of fat tailed degree distributions (30, 31). Empirical analyses of firm-level sectoral and aggregated European data using Stochastic Actor Oriented Models (SAOM) have emphasized the role of geography and as well as supplier heterogeneity in productivity, growth, labor costs and present, and present structural network properties on the evolution of production networks (40–42). A recent model (43) combines a generative network model for the network topology based on (44) with a diffusion model for the link weights based on (45). In terms of data, previous studies are either purely theoretical (30, 31), calibrated on sector-level input–output data (34, 42), focused only on single sectors (39–41), or fitted—at least partially—to macroscopic network properties such as the degree distribution of firm-level data (33, 38, 43, 46). In SI Table S1 in SI Text 1, we summarize the literature mentioned here for more clarity.

## Results

### SCN characteristics and reporting thresholds

We study the Hungarian SCN based on VAT data records from 2014 to 2022, see Materials and methods. Figure 1d shows the number of firms (blue), N(t), and connections (orange), L(t), active in each month, *t*. Both quantities show pronounced jumps from in July 2018 and 2020 that are caused by a successive lowering of reporting thresholds (above which a VAT payment enters the data), see Materials and methods. We name the periods alphabetically as indicated in Fig. 1d. The change of reporting thresholds introduces a systematic bias that we cannot correct for, and we separately analyze the three periods highlighted in Fig. 1d. We find an average $\bar{N}(A)=50,615$ and $\bar{L}(A)=127,274$ in period A, $\bar{N}(B)=129,835$ and $\bar{L}(B)=378,037$ in period B, and $\bar{N}(C)=282,879$ and $\bar{L}(C)=3,669,802$ in period C. Subannually both, *N* and *L*, show a seasonality, where the lowest numbers occur in January and the highest in December.

In Fig. 1e, we plot the average degree k(t)=L(t)/N(t) for every month. With lower reporting thresholds the network becomes denser, from $\bar{k}(A)=5.0$ in period A, $\bar{k}(B)=5.7$ in period B, to $\bar{k}(A)=25.9$ in period C. The most substantial densification occurs from B to C, suggesting a large number of low-transaction-value supply connections. The relative subannual change is smaller than for *N* and *L* in Fig. 1d, suggesting that the fluctuations in those quantities are driven by node activity.

### Firm- and link-turnover

Figure 1c shows the average relative annual change rates for the four processes described before in Hungary. Between 2015 and 2017 (period A) we find that per year about 25% of firms exit the Hungarian VAT network and 28% new firms enter; resulting in a net growth of 3.3%. On average, 55% of all links present in 1 year are not present the next year. However, relative to the previous year, also 61% of new links appear, resulting in an effective growth of 5.8%. The average link is found to have a half-life time of 13 months. These numbers indicate a massive, ongoing restructuring of the SCN. In SI Text 2, we investigate the link turnover rates by supplier and customer industry. We find very different link entry and exit rates depending on the specific supplier–customer industry combination.

### Estimation of entry and exit rates

We start by analyzing the entry and exit rates for firms and links, respectively, in the *persistent network*; for its definition, see Materials and methods. In Fig. S5a, we plot the empirical probability density function (PDF) of the monthly number of firms that enter the production network in 2017 (blue). The dashed vertical line denotes the empirical average of 348.9 new firms per month. The solid orange line shows the PDF of a superposition of Poisson processes that takes the annual seasonality in rates properly into account, see SI Text 3. Figure S5b shows the number of firm exiting per month in 2017 (blue). The dashed vertical line denotes the empirical average at 167.6 removed firms per month. In Fig. S5c and d, we plot the histogram of links entering and exiting per month in 2017, respectively. The dashed orange lines denote the averages at 867.8 for links entering and 606.9 for links exiting per month. All four processes, entry and exit of links and firms, can be modeled well with Poisson processes (orange lines). Here, we report overall firm and link turnover rates, not distinguishing whether a link has vanished due to firm exit or otherwise. We will distinguish these cases below.

### Firm entry

In the model, firms enter the network according to an Poisson distribution, with the mean rate matching the observed average monthly entry of new firms. Upon entry, each firm establishes connections with both suppliers and buyers. These connections are quantified by the firm’s in-degree (number of supplier links) and out-degree (number of buyer links). New firms in our dataset show an average in-degree of 0.35 and out-degree of 0.71. Notably, we observe a correlation between in-degree and out-degree, which we capture in our model by sampling from the empirical joint distribution illustrated in Fig. S8. The node’s sector is randomly drawn with the probability of sampling a sector proportional to its prevalence in the network,


(1) p(s)=n(s)/N,

where n(s) denotes the number of firms in sector, *s*, and *N* the overall number of nodes.

### Firm exit

The model implements firm exits in two distinct ways, calibrated to match the observed exit rates from our dataset. In the first mechanism, firms are selected for removal with a uniform probability $p^{\mathrm{ex}}$, along with all their incoming and outgoing connections. The second mechanism removes isolated firms – those without any connections – at the end of each timestep. The probability, $p^{\mathrm{ex}}$, is calibrated to match the empirical exit rate, $p^{\mathrm{exit}\;\mathrm{empirical}}$, using the relation


(2) $$p^{\mathrm{exit}\;\mathrm{empirical}}=p^{\mathrm{ex}}+\sum_{k}p(k)(p^{\mathrm{remove}\;\mathrm{link}}{)}^{k},$$

where the first term denotes the first uniform node removal probability and the second the probability of a node exiting due to removal of all its links. Together, these exit mechanisms successfully reproduce the left-skewed distribution of monthly firm exits observed in the data, as shown in Fig. S11a.

### Link exit

**Figure 2:**
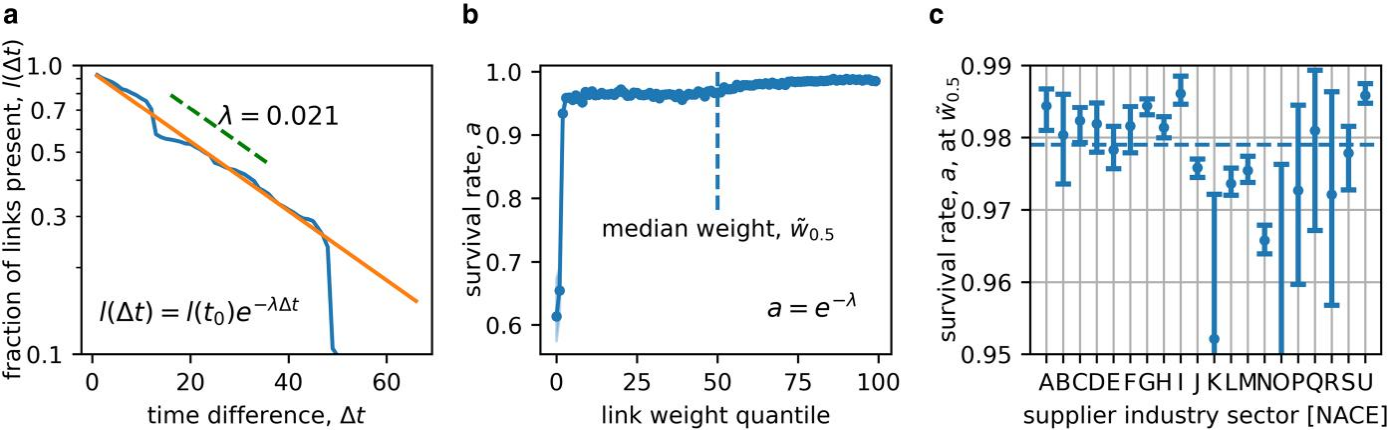
Estimation of link decay rates in the Hungarian SCN. a) To estimate the persistence of supply links, we identify all links, that exist at time $t_{0}=\mathrm{January}\;2017$ and plot the fraction, l(Δt), that is still active at time $t_{0}+\Delta t$, $l(\Delta t)=L(t_{0}+\Delta t)/L(t_{0})$. We fit an exponential decay to obtain the monthly decay rate. b) Survival rate, defined as $a=e^{-\lambda }$ as a function of link weight percentiles. The line denotes the average survival rate, the shaded area is the 90% CI. c) Survival rate for the NACE sections of the supplying firm at the median link weight. The dashed, horizontal line denotes the average overall survival rate of 95%. The link exit is well described by an exponential decay that has a lower rate for higher link weights and is heterogeneous for different industries.

In Fig. 2 we study the link exit process. To understand the persistence of supply links we identify all links at time $t_{0}=\text{January 2017}$, $L(t_{0})$, and plot the fraction of them that is still active at time $t_{0}+\Delta t$, $l(\Delta t)=L(t_{0}+\Delta t)/L(t_{0})$ (blue line). We fit an exponential decay $l(\Delta t)=e^{-\lambda \Delta t}$ (orange line). For $t_{0}=\text{January 2017}$, we find an overall (including all links) λ=0.021. This can be transformed to the survival rate, $a=e^{-\lambda }=0.979$ or the link removal probability of


(3) $$p^{\mathrm{remove}\;\mathrm{link}}=1-a=0.021.$$

This means 97.9% of all links that exist in one month also exist the following month and the yearly survival rate is thus 77.5%. This is significantly higher than reported in Fig. 1c because we are studying the dominant network, which is more stable than the overall change considered in Fig. 1c. We check if the link exit process can be described better by a process with a changing decay rate; see SI Text 4. We find that the data are best described by the process with constant decay rate.

How the survival rate (or decay constant) depends on the link weight (transaction volume), we show in Fig. 2b where the survival rate, *a*, is plotted as function of the link weight quantile, the shaded area denotes the 90% CI. Below the 20th percentile of link weights *a* is low, increasing from 0.614 for the lowest to 0.965 for the 20th percentile. Above the 20th percentile, we observe a formation of a plateau (with only a small increase) to 0.988 for the links with the highest volume. At the median link weight, ${\tilde{w}}_{0.5}=188\;\text{million Forint}$ (HUF, ∼480,000 EUR), we find $a_{{\tilde{w}}_{0.5}}=0.968$. Clearly, links with higher volume become more stable, above the bottom quintile the survival rates become practically constant; links in the bottom quintile are much more volatile and volume dependent.

To clarify the dependence of the survival rate on the industry cluster of the supplying firm, we fit the decay rate at median link weight for every supplier industry sector independently (using section level NACE (15) codes) and show the results in Fig. 2c (bars denote the 90% CI). The variability of *a* between sectors is high, with values ranging from $a_{O}=0.875$ in sector O to $a_{I}=0.986$ in sector I. See Table S2 for the descriptions of the NACE codes. There manufacturing sectors (B–F) tend to have higher survival rates than service sectors (G–U). For sectors with very few observations (as in K, O, P, Q and R), the CI is large. Sectors “I - Accommodation and Food Service Activities,” “A—Agriculture, Forestry and Fishing,” and “G - Wholesale and Retail Trade; Repair of Motor Vehicles and Motorcycles” are more stable than average, while sectors “N - Administrative and Support Service Activities” “K - Financial and Insurance Activities,” and “O - Public Administration and Defence,” appear to form relatively short lived connections. Note that for sectors N and O there are not many observations and the CI are large. In Table S4, SI Text 4, we provide a detailed table of sector level survival rates. The situation gets slightly more involved for link entries, where one has to distinguish between suppliers and customers.

### Link entry—customers

**Figure 3.**
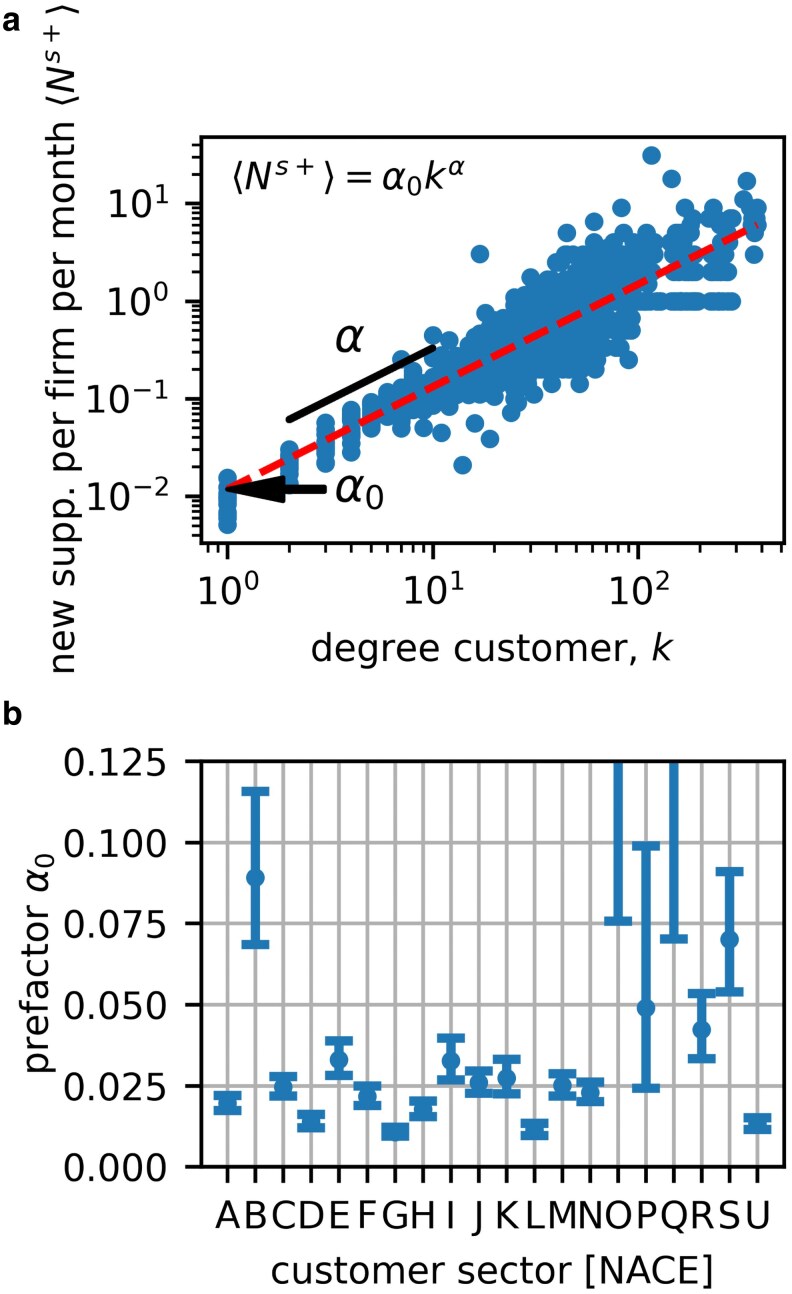
In-link generation overall and for industry sectors. a) Average number of new suppliers per firm per time step (month) as a function of the degree, $\left\langle N^{s+}\right\rangle$, for every degree and month in 2017. The dashed line shows the best fit line $\langle N^{s+}\rangle =\alpha_{0}k^{\alpha }=0.012k^{1.049}$. b) To assess differences in supplier-turnover, we fix the scaling exponent α=1.0 and fit the linear slopes, $\alpha_{0}$, for every NACE sector. Bars denote the 90% CI. For most industries the linear slope is between 0.01. and 0.04, only for industries with a very few (below ca. 50 observations, identical with those that show large errorbars) $\alpha_{0}$ is larger.

When links enter the network not all firms have an equal probability to appear as customers or suppliers. We first analyze link entry from the perspective of a customer by calculating the average number of new suppliers per firm per month, $\left\langle N^{s+}\right\rangle$. Later, we investigate to which firms these links attach. In Fig. 3a, we show $\left\langle N^{s+}\right\rangle$ as function of degree, *k*, and using OLS on the log-variables we fit a power function,


(4) $$\langle N^{s+}\rangle =\alpha_{0}k^{\alpha }$$

and obtain an approximate scaling relation. The fit (red dashed line) yields $\alpha_{0}=0.012\pm 0.002$ and α=1.049±0.027, where we report the 90% CI.

To estimate the baseline rate at which firms acquire new suppliers by sector, we fix the scaling exponent to α=1.0 and fit $\alpha_{0}$ separately for each customer industry. Fixing α=1.0 corresponds to assuming that (in a steady state) suppliers are replaced at the same rate by firms of all size. In Fig. 3b, we plot $\alpha_{0}$ by industrial sector using NACE sections, the error bars denote the 90% CI. For sectors with a few observations the error is large and results are not useful. Several sectors have turnover rates that are significantly larger than average, in particular (listing only sectors with small CI) “E - Water Supply; Sewerage, Waste Management and Remediation Activities,” I, and K. Sectors with significantly slower than average supplier turnover are “D - Electricity, Gas, Steam and Air Conditioning Supply,” G, and L. In Table S5 in SI Text 5, we list the $\alpha_{0}$ and *α* for all and time periods. Its instructive to compare the values for supplier turnover in Fig. 3b with the rates for customer turnover in Fig. 2c. Some sectors have high link-decay (as supplier) and high $\left\langle N^{s+}\right\rangle$ (as buyer), these sectors, for example I have high turnover up- and downstream. Sectors with low link-decay (as supplier) and high $N^{s+}$ (as buyer), for example E have stable customers, but switch suppliers often.

### Link entry—suppliers

**Figure 4:**
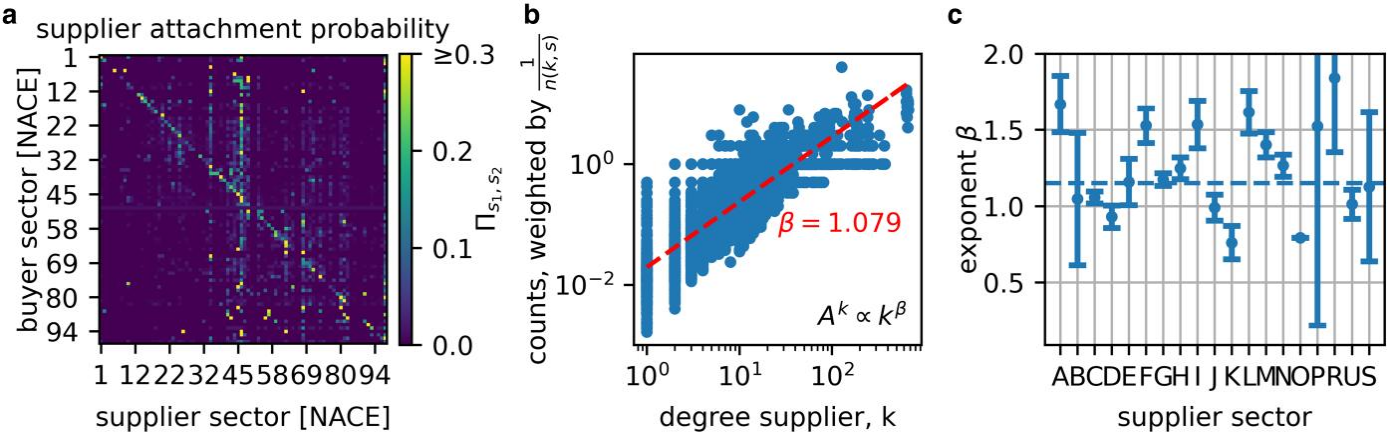
Estimation of the process how links attach to suppliers. We estimate the link attachment in two steps. We start with a) The supplier attachment probability matrix, $\Pi_{s_{1}s_{2}}$ for 2017, showing the probability that a firm in industry $s_{1}$ will choose a supplier from sector $s_{2}$. The matrix is clearly sparse, the diagonal and a few vertical lines are visible. b) Estimation of the attachment kernel exponent, *β*. To estimate the attachment kernel, $A^{k}(k)$, as defined in the main text, we plot the number of new out-links attaching to firms with a degree $k^{*}$ inversely weighted by $n(k^{*},s^{*})$. The dashed line shows the best fit to a scaling law $A^{k}(k)=\beta_{0}k^{\beta }=0.02k^{1.079}$. c) Scaling exponents, *β*, for the NACE sections, with 90% CI. The horizontal dashed line corresponds to the slope in b. The exponents show a lot of heterogeneity, ranging from 0.76 to 1.84, but are clearly larger than 0, highlighting preferential attachment.

Next, we characterize the new links from the supplier perspective. We do this in two steps. First, we analyze to what extent some sectors are more likely to link to each other by calculating the conditional probability for a customer in sector $s_{2}$ to link to a supplier in sector $s_{1}$, summarized in the *supplier attachment probability* matrix $\Pi (s_{1},s_{2})=p(s_{1}\;|\;s_{2})$, shown in Fig. 4a. High (low) linking probabilities are shown as light (dark) colors. The matrix is relatively sparse, meaning that most new links are focused on a few sector combinations only. The pronounced diagonal shows that firms are likely to link to firms in their own sectors. A few vertical lines are prominently visible, eg “G46 - Wholesale,” “D35—Electricity, gas, steam, and air conditioning supply,” and “L68 - Real estate activities.” These sectors are important suppliers to many other sectors. There are also two faint blocks visible, corresponding to manufacturing (1–43 or A–F, upper left) and service (45–99 or G–Z, lower right) sectors. The average within-block similarity is $\langle \Pi_{\left[A-F],[A-F\right]}\rangle =0.013$ and $\langle \Pi_{\left[G-Z],[G-Z\right]}\rangle =0.019$, the average off-block similarity $\langle \Pi_{\left[A-F],[G-Z\right]}\rangle =0.011$ and $\langle \Pi_{\left[G-Z],[A-F\right]}\rangle =0.004$. We test that the difference is statistically significant using a Mann–Whitney U test, rejecting the null hypothesis that the blocks are sampled from the same distribution (P<0.001). We use the nonparametric test because neither the diagonal nor the off-diagonal similarity values follow a Gaussian distribution.

Second, we ask how likely will a new link connect to a supplier with degree *k* in the selected sector, $s_{2}^{*}$? We answer this by estimating the function that determines the probability of attracting new out-links in a given sector. This function is only based on the current degree of the node, and we will call it the *attachment kernel*, $A^{k}(k)$. We measure it by adapting the method used in (47) and applying it to the monthly data in period A. The probability that a new link attaches to a node with degree *k* is now given by $P(k)=\frac{1}{\tilde{N}}A^{k}(k)n(k,s_{2}^{*})$, where n(k,s) denotes the number of nodes with degree *k* in sector *s*, and $\tilde{N}$ the normalization. The attachment kernel $A^{k}(k)$ can be estimated from a histogram where we count the number of new suppliers per degree bucket weighted by $\frac{1}{n(k,s)}$. We use linear buckets of size Δk=1. In Fig. 4b, we plot the number of new suppliers per degree bucket per month for period A, weighted by $\frac{1}{n(k,s)}$. We fit an attachment kernel of type $A^{k}\propto k^{\beta }$ using OLS on the log-variables and find slightly super-linear attachment, β=1.079±0.018, we report the 90% CI. In Fig. 4c, we fit $A^{k}$ for different NACE industries, unveiling a slight bias towards super-linear attachment with a few exceptions in C, D, J, K, O, and U, see also Table S6 in SI Text 6 for sector names and the results for the other time periods.

### Generative model for the evolution of SCNs

**Figure 5:**
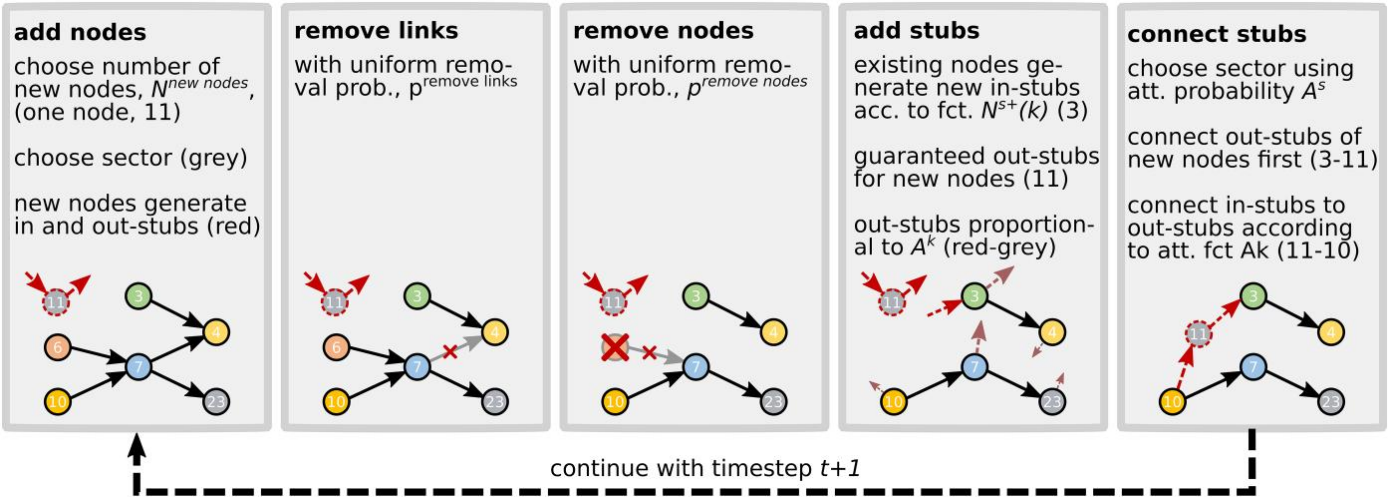
schematic view of the SCN generating model. It evolves from one timestep to the next in the following five steps: (1) add new firms. The number of firms is sampled from a Poisson process and the sector is drawn from the empirical sector distribution. New nodes are added to the network with a number of in- and out-links, $k^{\mathrm{in}}$ and $k^{\mathrm{out}}$ sampled from the empirical joint distribution, $p(k^{\mathrm{in}},k^{\mathrm{out}})$. (2) remove links with uniform probability, $p^{\mathrm{remove}\;\mathrm{links}}$. (3) remove firms with uniform probability, $p^{\mathrm{remove}\;\mathrm{nodes}}$. All their links are eliminated. We remove all isolated nodes that lost their links in this link elimination. (4) add stubs—spontaneous link creation. Existing nodes generate new in-stubs according to $N^{s+}(k)$. (5) connect firms. In stubs are connected by first choosing a sector to attach them to using the sector attachment probability, $\Pi_{s_{1}s_{2}}$, and then picking a node with probability that is proportional to the attachment kernel, $A^{k}(k)$. However, before connecting to nodes based on $A^{k}$, links are connected to out-stubs of new firms. This guarantees that firms keep attached to the SCN.

**Figure 6.**
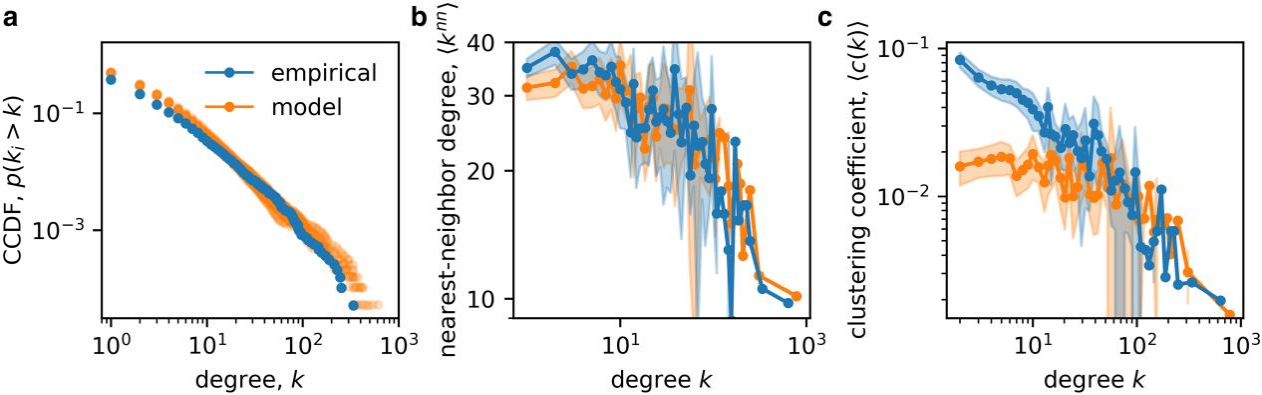
Model results of structural properties of SCNs. a) Counter cumulative degree distribution, $p(k_{i}\geq k)$ for snapshots generated by the supply network generating model and the empirical degree distribution in January 2017. The distributions are very similar. b) Average nearest neighbor degree, $\left\langle k^{nn}\right\rangle$, for the empirical and model networks using linear bins for k≤10 and log bins for k>10, the shaded area denotes the 90% CI. The disassortativity structure of both networks is practically identical. c) Average local clustering coefficient $\langle c_{i}\rangle_{i\;|\;k_{i}\in [k,k+\mathrm{bin}]}$ as a function of degree for the empirical and model networks (using same binning and error). The hierarchical structure reflected by the power law decay of the clustering coefficient as a function of the degree is again well captured especially for large degrees, but is somewhat underestimated for low degrees.

We are now in the position to use the previous estimates to calibrate a simple statistical generative model to the actual Hungarian SCN. The model is initialized with a set of firms (nodes), N, and a set of links, L. Here, we initialize the model with one snapshot of the empirical network, ie January 2017 in SI Text 7, we show that the model is robustness to different initializations. The network follows the topology shown in blue in Fig. 6 and contains 18,805 nodes. Note, this network is significantly smaller than shown in Fig. 1d, because here we use only firms and links in the “persistent network,” see Materials and methods. We initialize the model with the parameters shown in the first five rows of Table 1. We perform the following five steps at every timestep; for a schematically overview, see Fig. 5.

1. *Add new firms.* The number of new nodes is drawn form a Poissonian distribution with mean $N^{\mathrm{new}\;\mathrm{nodes}}$, as shown in Fig. S5a. Every node enters with an in-degree, $k^{\mathrm{in},0}$, and out-degree, $k^{\mathrm{out},0}$, that are sampled from the empirical joint distribution of in- and out-degree upon entering, $p(k^{\mathrm{in},0},k^{\mathrm{out},0})$, for details see SI Text 8. For every newly created firm, *i*, we draw its sector, $s_{i}$, from the empirical sector-distribution, p(s) as in (1).
2. *Spontaneous link removal.* With probability, $p^{\mathrm{remove}\;\mathrm{link}}$, (see (3)) every existing link in the SCN is eliminated, regardless of any features of the nodes or link weight.
3. *Firm removal.* Firms are removed with a uniform removal probability, $p^{\mathrm{ex}}$ (see (2)). All links connected to these firms are eliminated. We then also remove all those nodes that have become isolated after this link-removal step.
4. *Spontaneous link creation.* In- and out-stubs are added to every existing firms, *i*, that determine the number of new in- and out-links that the firm will acquire in this timestep. In-stubs represent to how many new suppliers each firm will connect to; out-stubs are proportional to the probability that an in-stub connects to a given supplier, *j*, ie its *attachment kernel*  $A^{k}(k)$. The number of in-stubs is drawn form a binomial distribution with mean $\left\langle N^{s+}\rangle (k_{i}\right)$, (see (4) in Link entry—customers section) as shown in Fig. 3. The out-stubs of every firm, *j*, are proportional to its *attachment kernel*, $A^{k}$, as calculated in Link entry—suppliers section and shown in Fig. 4b. Both quantities are not sector specific.
5. *Connecting the firms.* We connect every in-stub to a supplier in a two step process: (i) We choose the sector of the target firm, *j*, (supplier) with probability $\Pi_{s_{i},s_{j}}$, and (ii) then connect the in-stub to a firm in $s_{j}$ with probability, $p(j)=A_{j}/\sum_{k\in s_{j}}A_{k}$; see Link entry—suppliers section. The process ends when all in-stubs are connected to a supplier. To make sure that every new firm reaches its determined in- and out-degree, we connect to them first, until they have acquired $k^{\mathrm{in},0}$ and $k^{\mathrm{out},0}$ customers.

The only size dependent mechanisms are the ones determining in- and out-degree in steps 4 and 5. The only step depending on the firm’s sectors is when the stubs are connected in step 5. New nodes are added with predetermined size and sector, and both firm and node exit is not influenced by neither, size, nor sector.

**Table 1:** Model calibration and results.

		Period A		Period B		Period C	
		Empirical	Model	Empirical	Model	Empirical	Model
Calibration quantities							
Average number of firm entries	$\left\langle N^{\mathrm{entry}}\right\rangle$	357	=	605	=	904	=
Node exit probability	p(nodeexit)	0.0049	=	0.0051	=	0.0046	=
Intercept link generation function	$\alpha_{0}$	0.0108	=	0.0118	=	0.0160	=
Exponent link generation function	*α*	1.0369	=	0.9955	=	0.9711	=
Attachment kernel exponent	*β*	1.08	=	1.08	=	1.06	=
Link removal probability	$p^{\mathrm{term}}$	0.0214	=	0.0195	=	0.0338	=
Modeled quantities							
Number of nodes	*N*	20,059.9	18,339.9	33,971.6	33,700.6	41,817.2	41,646.6
Number of links	*L*	28,177.3	28,132.2	59,349.9	54,517.0	79,474.1	64,143.9
Average degree	⟨k⟩	2.81	3.06	3.49	3.24	3.80	3.08
Node volatility	${\hat{\sigma }}^{2}(N)$	704,671	36,067	682,905	198,381	2,825,670	398,510
Link volatility	${\hat{\sigma }}^{2}(L)$	2,412,146	1,558,618	3,858,995	894,264	19,535,266	1,057,220

The first part of the table shows the calibration values used for each time period (see Fig. 1).

Firm addition is followed by stochastic node removal calibrated to maintain a stationary network size; newly added firms are therefore, in principle, eligible for removal, but this ordering does not affect aggregate outcomes.

### Model results

While the number of firms in the real network is growing by 3.3% every year, we prefer to simulate a stationary economy and slightly increase the node removal probability p(node exit) such that we remove the same number of nodes that is added on average. In SI Fig. S9, we show the timeseries of number of firms and links.

We initialize the model with the empirical network topology of January 2017 and run it for 500 time steps. A timestep is calibrated to correspond to one month. After this initial phase, we take a snapshot of the model SCN at every 200 timesteps for further analyses. In total, we analyze 10 such snapshots. The model captures the network size dynamics with respect to the number of firms, *N*, and links, *L*; see Table 1. In SI Fig. S11, we show the monthly change of firms and links, ΔN and ΔL, respectively. Both quantities show a negative skew with a long negative tail; since we remove isolated nodes, the spontaneous exit of high-degree nodes can cause cascades of exits starting from its neighbors.

The firm- and link-volatility in the model as measured by the 12-month sample variance, $\sigma_{N}^{2}=36,067$ and $\sigma_{L}^{2}=1,558,618$, respectively, underestimates the empirical variance in both periods, which are ${\bar{\sigma }}_{N}^{2}=704,671$ and ${\bar{\sigma }}_{L}^{2}=24,121,146$, respectively. This is a consequence of the fact that model does not take empirical seasonality into account.

We continue with describing structural network features that emerge from the model. In Fig. 6, we show the degree distributions, $p(k_{i}>k)$ (CCDF), of the snapshots (orange) and compare them to the empirical degree distribution of January 2017 (blue). Note the agreement across almost 3 orders of magnitude. We find average degrees of $\langle k^{\mathrm{model}}\rangle =3.06$ and $\langle k^{\mathrm{emp}}\rangle =2.81$. The model also captures the differences the empirical in- and out-degree distributions; see SI Text 9.

SCNs are known to be disassortative, meaning that high-degree firms tend to be linked to low–low-degree firms and vice versa (17, 22). We confirm this assortativity structure in Fig. 6b by plotting the average nearest neighbor degree, $\langle k^{nn}\rangle =(1/k_{i})\sum_{\;j\in N_{i}}k_{j}$, as a function of degree *k*, where $N_{i}$ is the set of direct neighbors of *i*. The empirical network is the blue line, the ten model snapshots are the orange lines. The model reproduces the pronounced disassortative structure very well for all degrees.

A decaying clustering coefficient $c_{i}$, when plotted as a function of the degree *k*, indicates the presence of nontrivial hierarchy in complex networks (48). It is defined as $c_{i}=2t_{i}/k_{i}(k_{i}-1))$, with $t_{i}$ the number of triangles *i* is involved in. In Fig. 6c, we plot $\langle c_{i}\rangle_{i\;|\;k_{i}\in [k,k+\mathrm{bin}]}$ as a function of degree using bins described in the caption. The model appears to accurately reproduce the hierarchical structure of SCNs especially for large *k* but deviates for low degrees. This might be because the model lacks a mechanism for triadic closure as proposed by other authors (33, 34), which seems to be most important for low degree firms.

#### Systemic risk profiles

**Figure 7:**
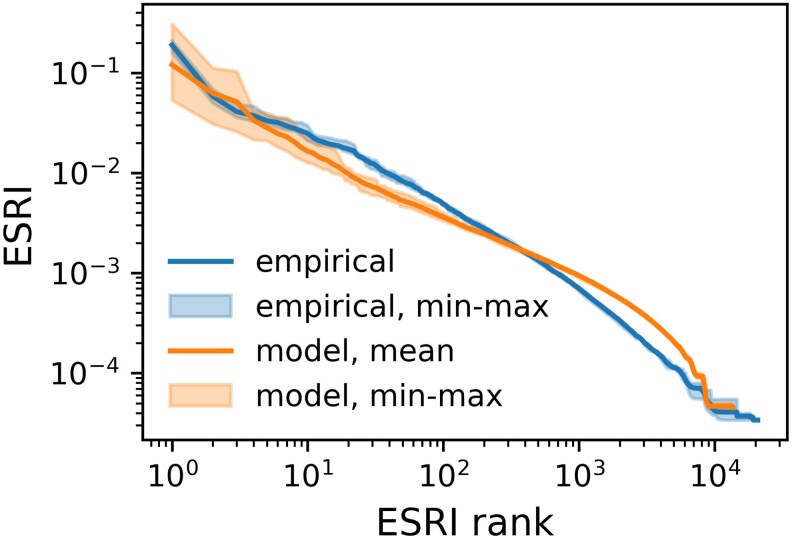
Economic systemic risk profile as measured with ESRI, from empirical data and 10 model networks from 10 snapshots. The ESRI value of every node is plotted against its ESRI rank. The empirical and model networks have remarkably similar systemic risk profiles across four orders of magnitude.

After having examined local network structures of the modeled SCN, we now turn to the question if the model also captures realistic structures that are relevant for spreading of defaults. For this, we turn to the Economic Systemic Risk Index (ESRI) as developed in (10). These structures are know to operate at a semilocal or mesolevel. The ESRI value of a firm quantifies the economic damage that its failure would immediately cause to the entire SCN, taking into account the network structure and production functions; for details see Materials and methods. ESRI is highly sensitive to the structure of the SCN, in particular correlations between network- and sector-structure (49). We calculate the ESRI for every firm in the empirical network of January 2017 and plot the rank-ordered distribution, from the highest to the lowest, in Fig. 7 (blue line). Note, here that the empirical network of stable connections exhibits no plateau of high systemic risk firms that was observed in annual data (10). We repeat the procedure for the ten model network snapshots and plot the mean ESRI for every rank (orange line) as well as the respective minimum and maximum ESRI values (orange shaded area). The ESRI of the 20 empirically most risky firms lies well within the min–max bounds of the model runs. For ranks higher than 20 the model underestimates the empirical ESRI, before crossing at around rank 400, where the empirical ESRI becomes smaller than the model estimate for the least risky firms. The network generative model manages to reproduce the ESRI well for the most risky nodes; however, the scaling exponent in the rank-ordered plot is somewhat lower than in the empirical network (from rank 10 to 1,000).

#### Results for different time periods

The model calibration was derived for period A. We perform the same calibration also for periods B and C and compare the results in Table 1 and SI Text 9.

We list the calibration quantities in Table 1. The parameters determining firm and link entry/exit rates ($\left\langle N^{\mathrm{entry}}\right\rangle$, p(nodeexit), $\alpha_{0}$, $p^{\mathrm{term}}$) are all higher in periods B and C, respectively, because the network is larger and features more small firms and links that are more volatile. We characterize the network with several key quantities. The model in periods B and C produces slightly sparser networks than empirically observed, as shown by 5 and 20% lower values for *L* and ⟨k⟩, respectively. The underestimation of the number of links and, consequently, the average degree, increases with the lowering of the reporting threshold, highlighting that when more volatile low-value links are included the sparse model calibration does not reproduce the full network structure. We also provide the variance of the *N* and *L* timeseries, ${\hat{\sigma }}^{2}(N)$ and ${\hat{\sigma }}^{2}(L)$, respectively. In all periods. For both timeseries, we underestimate the variances because we do not model the empirical seasonality.

## Discussion

We present a detailed statistical analysis of the four processes that govern the dynamical evolution of supply chain networks based on monthly VAT data from Hungary. We presented a minimum statistical model, in the sense that we try to use rates and marginal distributions whenever possible, and only employ joint distributions when unavoidable. This results in a network generative model that is calibrated with microdata and reproduces local structural network characteristics of the empirical SCN, including systemic risk profiles (that are based on nonlocal information).

We find rates of 2.1% node turnover (exit) and 4.6% link turnover (exit) per month. This means 25% (exit, 28% entry) node turnover and 55% (exit, 61% entry) per year. This is a somewhat unexpected result. While it is known that firms exit and enter at rates of 8% to 13% (50), the amount of turnover in links is less known and is striking. All of the economy is tremendously dynamical, it rewires completely every few years. This is an indication why it is resilient, it is able to reconfigure in responses to shocks because it is used to constant rewiring. Previous work has emphasized the relevance of the restructuring process of the SCN for the economic success and decline of firms (51), the resilience of production networks (52), and mitigating economic shock spreading (10, 53). The adaptive nature of SCNs has been emphasized (54). Our work contributes to this theoretical literature by providing an empirical description of the network restructuring processes. Moreover, our model explains how the observed substantial microlevel restructuring is consistent with stable macroproperties of the network, as described for example in (22). Here, we see our work in a tradition with the evolutionary economics view of industrial dynamics, in which persistent heterogeneity and selection operate within a stationary regime, rather than a stationary equilibrium (55).

We fit the node and link entry and exit rates with seasonally adjusted Poisson processes. This is found to describe the entry rates of firms and links well without considering sector or link-weight information. Of course, model results could be improved by incorporating the empirical heterogeneities in entry- and exit rates by sector and firm size, see SI Text 2. We focused on a minimal model, and see this as an avenue for future work.

We investigate the link exit process in more detail and find that the process is reasonably described by a constant decay rate, suggesting a process that is to first order memoryless, see SI Text 4. The decay rate depends on the link weight and varies strongly for links in the lowest weight quintile. This can introduce a bias in the network structure, because large link weights (sales) are typically associated with large firms—large sales in the network are orders of magnitude larger than those of small firms. The decay rate also varies strongly across the industrial sectors of the suppliers, reflecting the fact that time horizons vary for different economic activities.

We study link entry by characterizing both the customers and suppliers. The number of new suppliers per firm and per month can be described by a power law of the degree, $\langle N^{s+}\rangle =0.011k^{1.04}$, that is very close to scaling linearly with an exponent of 1. We find that the linear prefactor can be different for different sectors. This is intuitive, since as links get removed with a constant rate, larger firms need to generate new links proportional to their degree to maintain their size. After determining the number of new in-links a customer generates per month (changes of suppliers), we study the characteristics of the suppliers they attach to. We find that the supplier attachment matrix, containing the conditional probability for a customer in sector $s_{2}$ to attach to a supplier in sector $s_{1}$, $\Pi_{s_{1},s_{2}}$, is very sparse with the exception of the diagonal, ie the firm’s own sector, and a few “star” sectors, to which most other sectors frequently connect. These “star” sectors that supply to most other sectors include the NACE categories G46 (Wholesale), D35 (Energy), and L68 (Real estate).

In a given sector, firms tend to connect to large suppliers, a process well known as preferential attachment (56) (PA). We find super-linear preferential attachment with an average attachment kernel scaling exponent β=1.08. However, *β* can vary strongly between sectors. For example sectors “D - Electricity, Gas, Steam and Air Conditioning Supply,” “K - Financial and Insurance Activities,” and “O - Public Administration and Defence” feature pronounced sublinear PA, while “F - Construction,” “I - Accommodation and Food Service Activities,” and “L - Real Estate Activities” feature strongly super-linear PA. We find, however, clear evidence of preferential attachment across all industries with β>0 for every NACE section. Not including this sectoral heterogeneity will lead to wrong estimates of the tail exponents of industries.

We formulate and calibrate a simple network generative model that mimics the dynamics of a national SCN. As inputs it takes the rates and probabilities and probability distributions that were described so far. We test the model by comparing the structural network characteristics that emerge. We take snapshots of the emerging SCNs once in a stationary state and compare the derived characteristics directly with the empirical counterparts. The model is kept as simple as possible, ignoring the link-weight, sector, and seasonal dependency of the input parameters, whenever possible. Certainly, it possible to estimate link-weight, sectoral, and time dependent rates and probabilities in greater detail given enough data. Using these would then allow the model to capture the sectoral and seasonal network structure. This is subject to future work.

The generative model reproduces the number of firms as well as the in- and out-degree distributions. We find tail exponents of 2.653±0.087 and 2.620±0.76 for the modeled in- and outdegree distributions, respectively. These values are slightly higher, but of similar magnitude as the exponents of the empirical in- and outdegree distributions, 2.383±0.081 and 2.452±0.084, respectively (we report 90% CI intervals). Note, that we report significantly larger tail exponents than what is commonly found in the literature (22). This is due to the fact that we consider “stable” connections only, see Materials and methods. Previous models have not modeled the in- and out-degree distribution at the same time (38).

The model further reproduces the assortative mixing of the empirical SCN realistically, meaning that the network is disassortative, ie small firms tend to link to large firms and vice versa. The disassortative network structure is highly consistent across the literature (22) and distinguishes SCNs from other types of networks as for example social networks (27).

We find some deviations in the clustering behavior; for small degrees below twenty the empirical average local clustering coefficient is about a factor 2 larger than those from the model. Even though clustering coefficient is computed for undirected triangles, we checked whether the problem originates from the triangles where firms connect to suppliers of suppliers or firms connect to customers of their customers, but find no clear difference. This might arise from the fact that the model does not incorporate an explicit mechanism for triadic closure. In the literature the tendency to connect to suppliers of suppliers, which leads to closed triangles and higher clustering, has been proposed as a mechanism in SCN formation (33, 34). Our model misses this, because the attachment mechanism is based only on first-order properties of the node itself, not higher-order properties, such as the characteristics of a nodes’ neighbors. One could incorporate such mechanisms straight forwardly by making the attachment kernel depend not only on a node itself, but also on its neighbors.

Another potential explanation for the underestimation of the local cohesiveness is that our model does not consider the geographical proximity of firms, which is known to play an important role for supply link formation (22). If firms tend to connect to firms within their geographical region, reciprocal links and triads are more likely. Firms with many suppliers will have to source from outside their geographical region and the difference in clustering vanishes.

The model also is also able to reproduce the essence of the empirical *economic systemic risk profile*. There a few firms with a high ESRI value are observed (sytemic risk core; see Ref. (10)) and a power-law decay in the rank ordered ESRI distribution covering a similar range of values as reported in the literature (see SI Text 10 and Ref. (10)). This is somewhat unexpected, since ESRI is highly sensitive to correlations between network- and sector-structure (49).

Empirical and modeled ESRI values are of similar magnitude, their tails drop with a different slope in the rank ordered distribution. Note, that the empirical ESRI profile does not show a plateau of high systemic risk firms that was observed in yearly aggregated data (10, 27). The filtering to only stable links causes the high systemic risk core to fracture and no plateau emerges; for details, see SI Text 10.

In addition to our results on ESRI, we can also connect the estimated rewiring statistics to several dimensions of supply chain risk that have typically been established qualitatively (54). The link half-life and its heterogeneity across sectors and transaction-weight classes capture *relationship stability* (and thus the likelihood of supplier continuity), while the strong concentration of attachment to large suppliers and a small set of “star” supplier sectors relates to *concentration and bottleneck risk*. At the same time, high baseline rewiring rates indicate an inherent adaptive capacity of the network, which may enable reconfiguration after disruptions but can also imply operational instability (54).

To get a feeling for the relevance of overfitting, we also calibrate a generative model with the data from the other time periods. We find it robust with respect to network size and other parameter combinations; however, the model underestimates the number of links and, consequently, the network density. This is because for the later periods the number of new suppliers scales sublinearly. With uniform link removal probability, this means that large nodes loose more links than they can replace. Hence, for the later periods it would be more relevant to model the size dependent link removal rate, as shown in Fig. 2b. The sample variance of firms and supply links is underestimated because the model does not explicitly model seasonality.

While some model parameters change little between periods, for example the scaling exponent of the attachment kernel, *β*, others change significantly, for example the exponent of the link generation function, *α*. We associate these changes mainly with the changed reporting thresholds in the data. Note that changes could also partly be due to technological change or policy actions. We hope that future work will better explain how calibration parameters are related to external factors. Along this way, one avenue could be to compare Hungary to different countries. For example, for Estonia and Belgium in 2022 EUROSTAT reports annual firm birth (death) rates of 16.6% (25.1%) and 9.0% (5.2%), respectively. Both represent evidence of significant node turnover, but the difference between them will have a large effect on the model, and we expect the link formation parameters to vary between these two countries. We still expect that link-turnover rates are high, but country comparisons or natural experiments, such as policy changes, can reveal the dependence of the model parameters on technological and political differences.

We believe that the generative model can be used to simulate the effect of technological or policy changes on the network structure and resilience. For example, one could study effects of electric vehicle adoption on the network structure and systemic risk by setting a higher entry rate in the respective sector.

Earlier network generative models of production networks differ from ours in important ways. While previous approaches either relied partly on fitted parameters (38) or modeled firm entry and mergers to match emergent properties of the Japanese economy (43), our model derives all processes and parameters directly from microlevel data and simultaneously reproduces both in- and out-degree distributions of the Hungarian production network. Further details and a discussion of these differences are provided in SI Text 1.

Our study is subject to four obvious limitations. First, data timing is imperfect: transactions may not coincide with VAT payment dates. Although reporting rules require firms to declare transactions in the correct month, misreporting and trade credit, which can cause delays in payment of up to 120 days (57), can cause distortions in the sequence of link formation. We expect this effect to be small because if these reporting delays remain approximately constant, our parameters should not be affected. Second, to accurately model link weights we would need to consider production capacities of firms and consistency between in- and output quantities, according to firm’s production functions, which is beyond the scope of our current technical capabilities. Third, we only consider the network of *stable* supply links to ensure a clear meaning of the beginning and ending of a buyer–supplier relationship (with having physical production networks in mind). This means that we discard the role of volatile and short-term links that might be essential, such as investment goods that do not occur as regular payments, but as one-off purchases. Fourth, several microeconomic mechanisms for link formation have been proposed (30, 31, 33, 34, 40, 42, 53). Here, we study the “effective” outcome of these mechanisms only. For example, the economic processes underlying preferential attachment include (among others) the fact that only some suppliers produce the products needed (53), the social network underlying the formation of business ties (33, 34), and of course price differences (31). The situation might become more transparent when more data on the involved firms could be included, such as geography, productivity, sales, etc. (58), which is impossible for us at this stage. In future work, higher order processes could be added to the model, such as triadic closure, firm splitting or merging, or introducing explicit seasonality.

Data of national supply chain networks on the firm level remain to hot topic (14). Typically, it is highly protected and can not shared freely. We see the value of this work not only in a comprehensive descriptive statistics showing a massive turnover in the economy, both in terms of firms and their supply links, but also to make a “digital twin” available to a wider community in form of the presented model; for the source code, see https://github.com/treisch/scn_generative.

## Materials and methods

### Data

We use VAT data provided by the Hungarian central bank. Since 2014 Hungarian firms need to report their suppliers with whom they exceeded a threshold of 1M HUF tax content in the reporting period (period A). In July 2018, the threshold was lowered to 100,000 HUF tax content and reporting is evaluated at the invoice level (period B). In June 2020, the reporting threshold was set to zero and firms needed to report all partners and invoices, irrespective of transaction volume (period C). We conducted robustness checks by retroactively imposing the highest reporting threshold throughout the sample period; however, the reporting changes in mid-2018 and mid-2020 remain clearly visible. We attribute this to the shift from thresholds applied to aggregated monthly totals to thresholds applied at the invoice level, which appears to have induced changes in firms’ reporting behavior. In 2014, the first year of granular VAT reporting in Hungary, data quality is poor and we do not consider this year in the analysis. The data contain information on transactions aggregated to monthly, quarterly, or annual levels (based on the firms exceeding certain reporting thresholds, see SI Text 11), as well as information on firm industry and size. We focus on monthly reported transactions which comprise on average 85.6% of the reported production volume.

### Stable supply links

For our analysis, we are interested in the *persistent network*, consisting of stable supply connections and not one-time purchases, even though these might be relevant. We define the existence of a supply link between two firms if there occur at least three transactions within a time window of six consecutive months, see SI Fig. S19 in SI Text 12. The time of a link-entry is the month of the first transaction that occurs in a time period where this condition is fulfilled, for the subsequent timesteps (months) we mark the link as “existing,” the time of link-exit is the month following the month in which the last transaction took place (where the condition is satisfied). The procedure removes about 77.9% of all links in period A, which accounts for about 18.5% of the overall purchase volume. The time a firm enters the SCN is recorded as its first overall occurrence; the time a firm exits by its last overall occurrence.

### Economic systemic risk index

We employ the systemic risk measure, ESRI, as described in Ref. (10). For every firm, *i*, it measures the immediate relative reduction of production in the SCN as a consequence of the firm *i*’s default. The algorithm requires the SCN information, an estimate of the production functions, and one has to specify the relative “essentialness” of sectors, for which we use estimates based on a survey among industry experts reported in Refs. (59) and (49). We use two-digit NACE codes for the industries. Note that here we do not consider link weights or the cost/revenue correction as in previous works. In SI Text 10, we analyze the effects of these parameter choices. The source code for ESRI is available https://github.com/ch-diem/misestimation_from_aggregation.

## Supplementary Material

pgag091_Supplementary_Data

## Data Availability

The raw data underlying this article are in part highly sensitive and cannot be shared. The derived data that is used to calibrate the model is available alongside a Julia implementation on https://github.com/treisch/scn_generative. The source code for ESRI is available https://github.com/ch-diem/misestimation_from_aggregation.

## Preprints

A preprint version of this article was posted on Arxiv https://arxiv.org/abs/2503.20594.
