# Supplementary material for: Supply chain network rewiring dynamics at the firm level
Source: PNAS Nexus. 2026 Apr 16;5(4):pgag091. doi: 10.1093/pnasnexus/pgag091 (PMC13126663; doi:10.1093/pnasnexus/pgag091)
Supplement: pgag091_Supplementary_Data [file pgag091_supplementary_data.pdf]

Supply chain network rewiring dynamics at the firm-level - Supplementary Information

Tobias Reisch,[a] András Borsos[a,b] and Stefan Thurner[a,c,d,e,*]

[a]Complexity Science Hub Vienna, Vienna A-1080, Austria, [b]Department of Financial Systems Analysis, Central Bank of Hungary, Budapest 1013, Hungary, [c]Section for Science of Complex Systems, CeMDAS, Medical University of Vienna, Vienna A-1090, Austria, [d]Santa Fe Institute, Santa Fe, NM 85701, USA and [e]Supply Chain Intelligence Institute Austria, Vienna A-1080, Austria

[*]To whom correspondence should be addressed: stefan.thurner@meduniwien.ac.at

© The Authors. All rights reserved.

SI Text 1: Literature overview table

In SI Tab. S1 we summarize the literature referenced in the main text. We list network generation models that work in a similar fashion to our model, with node and link additon and removal steps, respectively. We also include several studies that use firm-level supply network data to describe network restructuring processes, for example [4] proposes triadic closure as a process in production network formation. The studies using Stochastic Actor Oriented Models (SAOM) [10, 2, 3] highlight especially the role of geography and a range of other firm- and network-characteristics. In empirical analysis of the input-output structure of the European Union using a SAOM, *Mundt* [10] tries to synthesize several previous studies and finds that supplier heterogeneity in productivity, growth, labor costs, and several structural network properties –such as firm degree, the tendencies to connect to indirect suppliers, and to form reciprocal relationships– all play a role in the evolution of production networks. Earlier studies based on SAOMs were used to study the role of geography on the formation of firm ties [2, 3]. Finally, we include two theoretical contributions that first emphasized theoretical processes leading to the formation of fat-tailed degree distributions in a General Equilibrium setting. In the first study, a model is proposed in which firms adopt new suppliers based on price differences, providing an explanation for the Zipf-type distribution of firms' out-degrees [8]. In a more theoretical exercise, a model was studied where suppliers are selected based on both the match-specific productivity and the cost of the associated input, resulting in the emergence of "star suppliers" with a large number of customers [11]. The table highlights that our model captures a wide range of output quantities, while simultaneously being the only one fully calibrated from microdata. Further, our model is unique sonce it is calibrated with monthly VAT data.

Two network generative models for nation-wide production networks were developed previously. Reference [1] deals with the question whether the buyer–supplier network of the US economy is purely scale-free. They derive model parameters mostly from micro-data, but fit one last parameter to the in-degree distribution using a maximum likelihood estimator. In comparison, in our present model all involved processes and all parameters are derived and quantified on the micro-level, there are no free parameters. Further, our model simultaneously reproduces the in- and out-degree distribution of the Hungarian economy, compared to only the in-distribution in [1].

In [12] a two-layer model is proposed for the Japanese production network, combining a generative network model for the network topology with a diffusion model for the link weights. Only firms that enter the network form new links using preferential attachment; established firms grow by merging with other nodes. Model parameters are fitted to the emergent network properties such as the degree distribution, firm degree-growth rates, and the sales growth rate. In the presented model we take the opposite direction, by directly investigating firm behavior on the microscopic level and using a generative model to understand emergent properties which can be immediately compared with the empirical data. With our model we cannot confirm the results of [12] because, for example, their model describes the data best if 37% of firms are subject to mergers, and –due to a model assumption– the same fraction of is also subject to splitting. In Hungary these rates are much lower with values around 4 % in the years between 2015 and 2021.

Table S1. Tabular summary of the literature on supply network rewiring models referenced in the main text.

Reference	Description	Modelled quantities	Data	Temporal resolution	Comment
Atalay et al. [1]	Network generative model	Indegree distribution	Publicly listed firms (Compustat)	Annual	Most similar to our study; parameters mostly calibrated from microdata, only one parameter is fitted to the degree distribution with an MLE estimator
Ozaki et al. [12]	Combination of a generative network model [9] and a diffusion model for the link weights [15]	Degree distribution, link weights/sales	Japanese Corporate Credit Report database (provided by Teikoku Databank, Ltd.)	Annual	Link formation driven by node merging and splitting; model fitted to the macroscopic properties (degree distrbution, growth rates,...); we cannot confirm their results, for example they have 37% of firms merging and splitting every timestep
Saavedra et al. [14]	Generative model for declining networks	Degree distribution, LCC size, disassortativity	Collaborations in the NY garment industry	Annual	Preservation of asymmetric links (wrt. degree) plays large role
Chaney [5]	Network generative model for exporters with target locations	Outdegree distribution, outdegree per target market	French firm exports	Annual	Firms can only enter a foreign market if they have a contact there! (i.e. Triadic closure); model adapted from [1]
Carvalho & Voigtländer [4]	Model of technolocial adoption with implications for production network formation, regression based	Technological adoption matrices	Publicly listed firms (Compustat)	Annual	Not really a network generative model, but mentioned here because it first proposes the mechanism of triadic closure.
Mundt [10]	SAOM with geography and network characteristics	Indegree, outdegree	European sector level input-output data	Annual	SAOM's are a (temporal) alternative to ERGM's
Balland [2]	SAOM focussing on role of geography	Indegree, outdegree	Single sector, firm-level cooperation data (gobal navigation satellite system industry)	Annual	first study using SAOMs to highlight the relevance of proximity in network formation
Balland et al. [3]	SAOM focussing on role of geography, network characteristics across the lifecycle of an industry	Indegree, outdegree	Single sector, firm-level co-production (video game industry)	Annual	five types of proximity, study six generations of video games that show the rise and decline of the industry
Gualdi & Mandel [8]	General equilibrium coupled with network rewiring driven by price differences	Indegree, outdegree, instrength, sales, profits	no data, refer to stylized evidence of "Zipf-type degree distributions"	-	Purely theoretical, reproduces only stylized facts
Oberfield [11]	Network model with focus on firm's production techniques	Fat tailed outdegree distribution, emergence of "star suppliers"	no data, purely theoretical	-	Purely theoretical, reproduces only stylized facts
This study	Network generative model	In- & outdegree distribution, assortativity structure, clustering structure, sectoral structure, Economic Systemic Risk Index	Value Added Tax data	Monthly	only network generative model with all parameters calibrated directly from the microdata

SI Text 2: Disaggregated turnover

Industries organize their supply chains in different ways and turnover rates for links are not the same for all sectors and firm sizes. Here, we discuss entry and exit rates by supplier and customer sector and size.

In Fig. S1a we show relative link entry (blue) and exit (orange) rates by supplier sector for period A. The sectors with the highest entry rates are NACE sections Q, P, F, K (see Tab. S2 for sector descriptions); the sectors with the highest exit rates are sectors Q, P and K. For most supplier sectors entry rates are higher than exit rates, because the entire network is growing. Net growth is highest for B, G and M. In Fig. S1b we plot the relative link entry (blue) and exit (orange) rates in period A by customer sector. The sectors with the highest entry rates are K, P, and F; the sectors with the highest exit rates are K and P. For all customer sectors except NACE section K we observe net growth, with the highest growth rate for B, H and F. On average, manufacturing sectors (<G) have lower turnover rates than service sectors (>G), with the exception of sector "F - Construction".

We further characterize the link turnover by calculating exit and entry rates for each supplier-customer sector combination. Figure S2a shows the relative link entry rates for period A by supplier and customer NACE section. The entry rates are very heterogeneous, spanning values from 0 to 1. Very high or low values are typical for sector combinations with few observations. The matrix is not symmetric and entry rates can be much higher in one direction than in the other, the mean absolute difference for reciprocal pairs is 0.056. In Fig. S2b we plot the relative link exit rates for period A by supplier and customer NACE section. Again, the entry rates are very heterogeneous, spanning values from 0 to 1, with very high values typical for sector pairs with few links. The matrix is not symmetric and exit rates can be much higher in one direction than in the other, the mean absolute difference for reciprocal pairs is 0.102. For both entry and exit the top left corner, denoting the turnover rates between manufacturing sectors (except F, i.e. <F), shows lower values than if service sectors are involved.

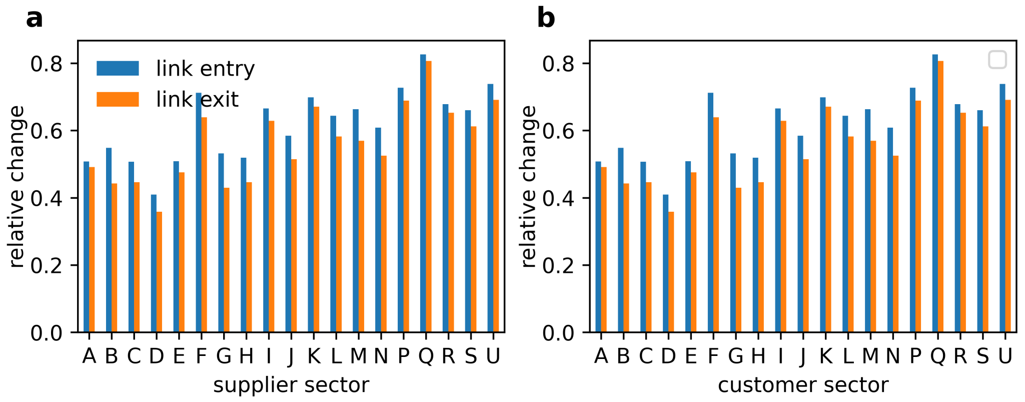

SI Fig. S1. Link turnover in period A by supplier and customer industry. Relative link entry rates are shown in blue and exit rates in orange by (a) supplier sector and (b) customer sector. Turnover rates vary strongly between industries and are lower for manufacturing sectors except construction (A-E) than for service sectors (I-U).

In Fig. S3a we plot relative link entry and exit rates as function of supplier strength, using logarithmic bins. Firms with low strength have generally very high customer turnover rates around 80%, but above strength values around 10^5kFT both relative entry and exit rates fall monotonically to around 30% for the largest firms. As the network is growing, entry rates are larger than exit rates for most strength values. In Fig. S3b we show the same plot for customer strength. Firms with the lowest strength values have very high supplier turnover rates around 90%, but both relative entry and exit rates fall monotonically to around 30% for the largest firms. As the network is growing, entry rates are larger than exit rates for most strength values.

We further characterize the link turnover for each supplier-customer size combination. Figure S4a shows relative link entry rates for period A by supplier and customer strength bin (logarithmic bins). The entry rates decay with higher strength of the involved parties. In Fig. S4b we show the same plot for relative link exit rates. Again, exit rates decay with higher strength of the involved parties. For both rates, there are few data points for low and high strength values and, hence, the entry and exit rates are noisy.

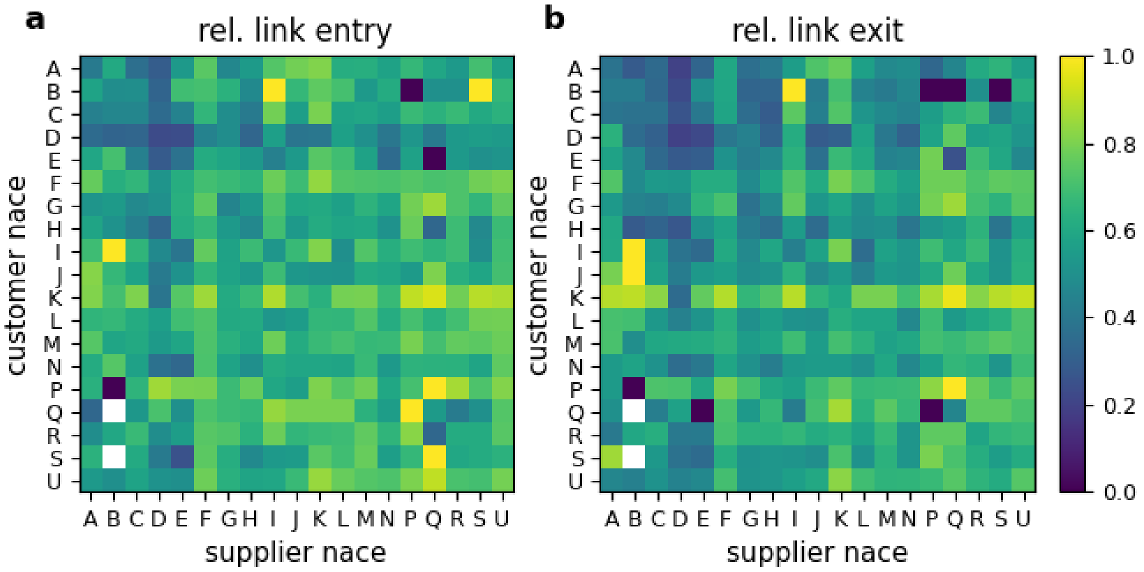

SI Fig. S2. Link turnover in period A by supplier and customer industry combination. (a) Relative link entry rates by supplier (column) and customer (row) industry. (b) Relatie link exit rates by supplier (column) and customer (row) industry. The values are very heterogeneous even for single sectors.

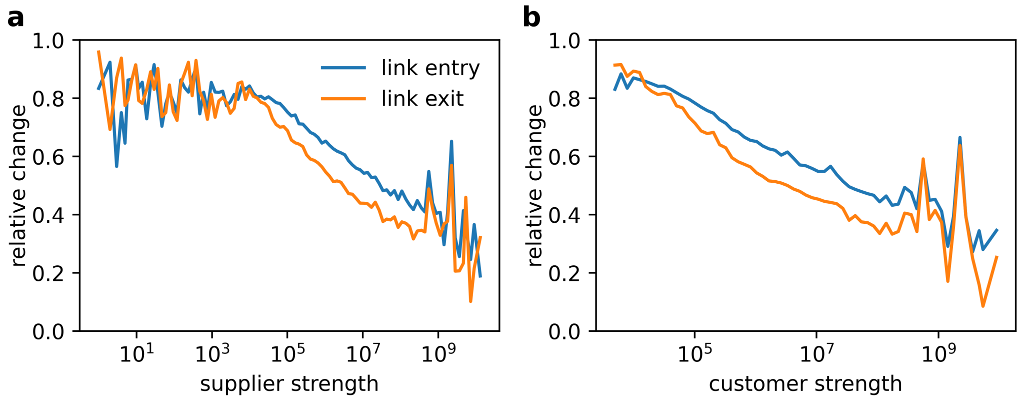

SI Fig. S3. Link turnover in period A by supplier and customer strength. (a) Relative link entry (blue) and exit (orange) rate as function of the supplier strength, values are calculated using 100 logarithmic strength-bins. (b) Same as in (a) but as a function of customer sector. In both panels we only plot bins that contain more than 100 links. Turnover drops after a threshold of around 10^5kFT and is lower for large firms, both on the supplier and customer side.

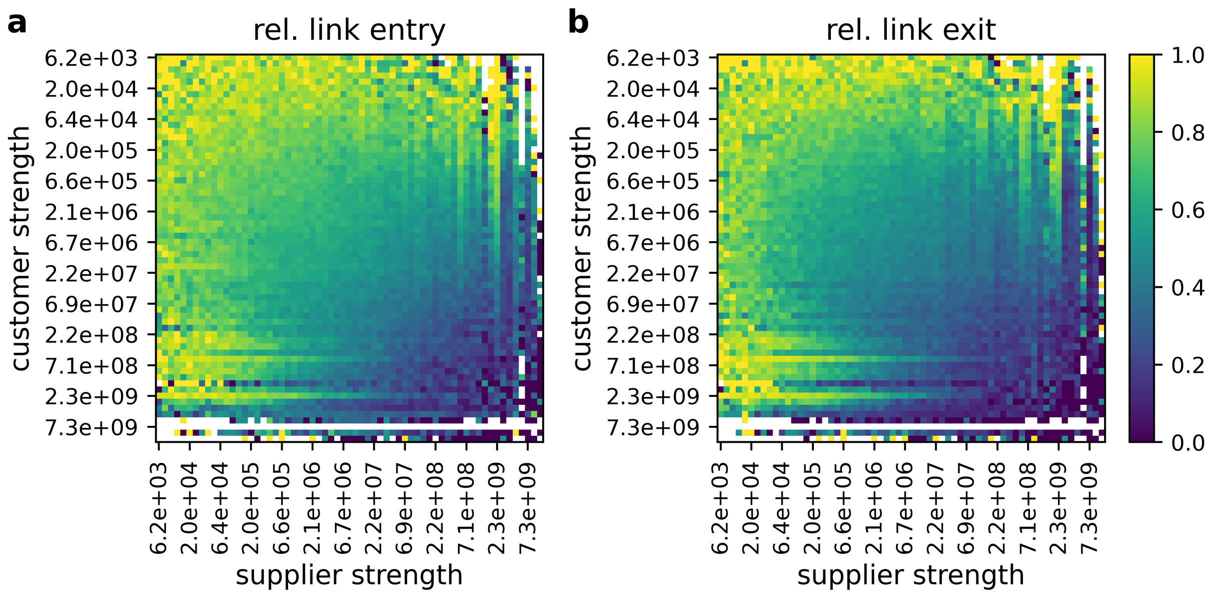

SI Fig. S4. Link turnover in period A by supplier and customer strength, using logarithmic bins. (a) Relative link entry rates by supplier (column) and customer (row) strength. (b) Relatie link exit rates by supplier (column) and customer (row) strength. Turnover rates decay with higher strength of the involved parties. For low and high strength values there are few data points and the entry and exit rates are noisy.

Table S2. Descriptions of NACE section codes.

Code	Economic Area
A	Agriculture, Forestry and Fishing
B	Mining and Quarrying
C	Manufacturing
D	Electricity, Gas, Steam and Air Conditioning S...
E	Water Supply; Sewerage, Waste Management and R...
F	Construction
G	Wholesale and Retail Trade; Repair of Motor Ve...
H	Transportation and Storage
I	Accommodation and Food Service Activities
J	Information and Communication
K	Financial and Insurance Activities
L	Real Estate Activities
M	Professional, Scientific and Technical Activities
N	Administrative and Support Service Activities
O	Public Administration and Defence; Compulsory ...
P	Education
Q	Human Health and Social Work Activities
R	Arts, Entertainment and Recreation
S	Other Service Activities
T	Activities of Households as Employers; Undiffe...
U	Activities of Extraterritorial Organisations a...

SI Text 3: Entry and exit rates

We analyze monthly entry and exit rates for firms and links, respectively. The time a firm enters the network is denoted by its first overall occurrence and the time a firm exits by its last overall occurrence. A link exists if it is present in three or more months in a six month window. It enters on the first month of the first window where this condition is fulfilled and exits on the first month after the last window where this condition is met.

We fit a Poisson distribution with a seasonality adjusted rate, using the following procedure. First, we count the number of monthly entry or exits, respectively, $X(t)$. Then we fit a linear trend to account for seasonality, $x(t) = \beta_1 \text{month}_t + \beta_0$, where month_t denotes the number of the month, starting with 1 for January and ending with 12 for December. The seasonality adjusted Poisson distribution is the mixture of twelve Poisson processes centered on $x(t)$,

$$p(X) = \frac{1}{12} \sum_{\tau=1}^{12} P_{x(\tau)}(X) \quad ,$$

where $P_\lambda(X)$ denotes the Poisson distribution with rate λ.

In Fig. S5 we show the seasonality adjusted entry rates as solid orange lines. The entry processes in panels a and c are described well. For the exit processes in panels b and d, respectively, the symmetric seasonality adjusted Poisson distribution fails to capture the skew of the empirical distribution. In Tab. S3 we report the average entry and exit rates for firms and nodes, respectively, separated by time period.

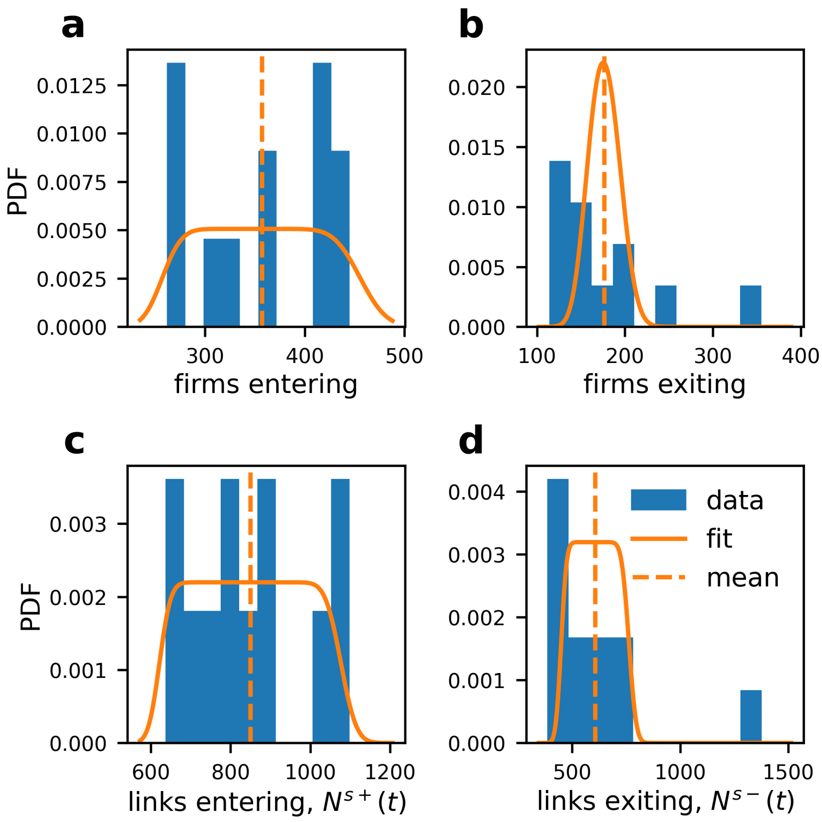

SI Fig. S5. Monthly entry and exit rates in the Hungarian PNW. (a) Empirical number of new firms per month (blue) in 2017, compared with a seasonality adjusted Poisson distribution (orange). The dashed vertical line denotes the empirical average new firms per time step, 348.9. (b) Empirical number of firms exiting per month (blue) in 2017, compared with a seasonality adjusted Poisson distribution (orange). The dashed vertical line denotes the empirical average number of removed firms per time step, 167.6. (c) Histogram of the empirical number of new links entering per month (blue) in 2017, compared with a seasonality adjusted Poisson distribution (orange). The dashed vertical line denotes the empirical average number of new links entering per time step, 867.8. (d) Empirical number of links exiting per month (blue) in 2017, compared with a seasonality adjusted Poisson distribution (orange). The dashed vertical line denotes the empirical average number of removed links per time step, 606.9.

Table S3. Table with average entry/exit rates, separated by time period.

	Period A		Period B	
	entry	exit	entry	exit
links	867.8	606.9	1697.3	1301.5
nodes	348.9	167.6	605.1	360.2

SI Text 4: Details on link decay

In this SI Text we provide details on the link decay process, in particular we discuss the functional form of the empirical link decay and we provide detailed results on sectoral decay rates for different time periods.

In the main text, we characterize link exit by counting the number of links present in the network at a time t, $L(t)$, and then counting again after a time Δt, $L(t + \Delta t)$. In Fig. S6a we plot the relative number of links that remains Δt months after $t_0 = 01/2017$, $l(\Delta t) = L(t_0 + \Delta t)/L(t_0)$. The relative fraction of links after Δt decays with a decreasing rate over time, but it is hard to tell the functional form of $l(\Delta t)$ from Fig. S6a.

In the main text we chose an exponential fit to describe the link decay process. This corresponds to a memoryless process with constant decay rate that has the differential equation $l'(t) = -cl$, which integrates to the exponential function $l(t) = ke^{-ct}$. Such a process manifests itself in a straight line in a semi-logarithmic plot, see Fig. S6b, and describes the data well.

Alternatively, one could propose a process with memory, i.e. where the decay rate decays over time. The differential equation for such a process, $l'(t) = -(c/t)l$ is solved by the power law $l(t) = kt^{-c}$, which would show up as a straight line in a double logarithmic plot. In Fig. S6c we plot the the data (blue) on a double logarthimic axis and the exponential fit (orange). Both the fit and the data are clearly not a straight line, suggesting that a scaling law describing a process with memory is not a good description for links in the Hungarian PN.

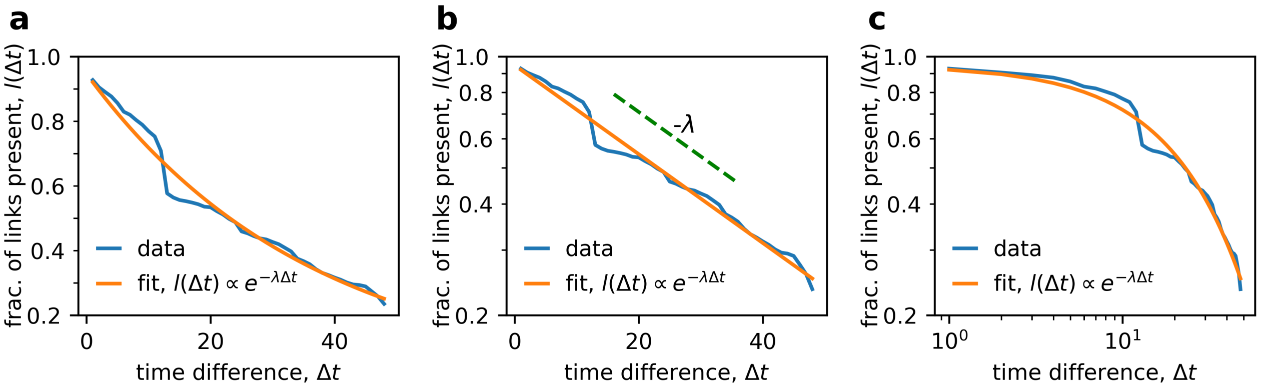

SI Fig. S6. Relative fraction of links present Δt months after Jan. 2017, $l(\Delta t)$, plotted using differently scaled axes. We plot both the data (blue) and the best fit (orange) using (a) linear, (b) semi logarithmic, and (c) double logarthmic axes. If the data follows an exponential decay, it plots as a straight line in the semi-logarthmic plot, if it follows a power-law decay it plots as a straight line in the double logarithmic plot. The exponential fit describes the data best.

In the main text we plot the decay rates for period A, In Tab. S4 we note the decay rates, $a_{\bar{w}_{0.5}}$, fitted at median weight, $\bar{w}_{0.5}$, for different time periods and NACE industries.

Table S4. Table with link decay rates, fitted at median weight.

	2017			2019		
nace	mean	5%	95%	mean	5%	95%
A	0.900995	0.885216	0.922171	0.900995	0.885216	0.922171
C	0.944346	0.940052	0.950722	0.944346	0.940052	0.950722
D	0.960553	0.956868	0.963245	0.960553	0.956868	0.963245
E	0.884572	0.799833	0.935946	0.884572	0.799833	0.935946
F	0.929888	0.917493	0.939161	0.929888	0.917493	0.939161
G	0.935416	0.933342	0.938008	0.935416	0.933342	0.938008
H	0.954000	0.951109	0.957627	0.954000	0.951109	0.957627
J	0.937026	0.930569	0.943492	0.937026	0.930569	0.943492
K	0.909709	0.865296	0.968903	0.909709	0.865296	0.968903
L	0.944099	0.941322	0.947060	0.944099	0.941322	0.947060
M	0.949814	0.946932	0.953335	0.949814	0.946932	0.953335
N	0.948567	0.944409	0.953664	0.948567	0.944409	0.953664
R	0.935168	0.914068	0.969111	0.935168	0.914068	0.969111
S	0.925820	0.925820	0.925820	0.925820	0.925820	0.925820
U	0.937560	0.934298	0.943567	0.937560	0.934298	0.943567

SI Text 5: Details on the average number of new suppliers per month

- Add α at least in the baseline estimation for every time period!!

In the main text we fit a scaling relation $\langle N^{s+}\rangle = \alpha_0 k^{\alpha}$ for the average number of new suppliers per month, $\langle N^{s+}\rangle$. For all periods the scaling exponent, α, is not statistically significantly different from 1. We fix $\alpha = 1$ and show the fitted $\alpha - 0$ for all NACE sections and time periods in Tab. S5. We report the 90% CI.

Table S5. Baseline supplier generation rate, α_0, for NACE sections and time periods. We report the 90% CI.

	2017			2019		
nace	α_0	5%	95%	α_0	5%	95%
A	0.02042	0.01813	0.02300	0.02027	0.01817	0.02261
B	0.09087	0.06955	0.11871	0.04608	0.03697	0.05744
C	0.02631	0.02330	0.02971	0.02129	0.01906	0.02377
D	0.01517	0.01306	0.01762	0.01758	0.01508	0.02049
E	0.03454	0.02939	0.04060	0.03430	0.02930	0.04017
F	0.02432	0.02115	0.02798	0.02552	0.02249	0.02896
G	0.01151	0.01015	0.01305	0.00996	0.00888	0.01117
H	0.01951	0.01703	0.02235	0.01808	0.01596	0.02049
I	0.03672	0.03006	0.04486	0.03072	0.02590	0.03643
J	0.02664	0.02328	0.03048	0.02532	0.02216	0.02892
K	0.02911	0.02402	0.03529	0.03142	0.02489	0.03966
L	0.01207	0.01019	0.01430	0.01350	0.01147	0.01589
M	0.02564	0.02234	0.02942	0.02336	0.02048	0.02664
N	0.02449	0.02143	0.02798	0.02162	0.01902	0.02458
O	0.20000	0.04196	0.95334	0.16758	0.09007	0.31181
P	0.06404	0.03514	0.11672	0.06021	0.03235	0.11204
Q	0.21064	0.08507	0.52154	0.07282	0.03417	0.15520
R	0.04672	0.03661	0.05963	0.05025	0.03964	0.06369
S	0.05981	0.04642	0.07706	0.05668	0.04361	0.07367
U	0.01434	0.01252	0.01643	0.01403	0.01233	0.01597

SI Text 6: Attachment kernel details

In the main text we fit a scaling relation $A^k \propto k^{\beta}$ to determine the attachment kernel exponent, β. In Tab. S6 we report β for all NACE sections and time periods, with the respective limits of the 90% CI.

Table S6. Attachment kernel scaling exponents, α, by sector and time period. We report the respective limits of the 90% CI.

	2017			2019		
	β	5%	95%	β	5%	95%
A	1.638276	1.458471	1.818080	1.369997	1.257529	1.482465
B	1.225872	0.829516	1.622228	0.860360	0.732422	0.988298
C	0.916849	0.869831	0.963868	0.958597	0.927150	0.990044
D	0.926334	0.852935	0.999734	0.823391	0.735633	0.911149
E	1.004818	0.835834	1.173803	1.000170	0.845342	1.154999
F	1.467235	1.348559	1.585911	1.484358	1.408171	1.560544
G	1.263201	1.210515	1.315888	1.212597	1.176773	1.248420
H	1.127075	1.035660	1.218490	1.071110	1.000068	1.142152
I	1.555724	1.397347	1.714101	1.579479	1.362083	1.796875
J	0.777848	0.682641	0.873056	0.843571	0.757417	0.929725
K	0.807811	0.687261	0.928360	1.080271	0.979805	1.180737
L	1.632160	1.493078	1.771243	1.468013	1.377034	1.558992
M	1.490059	1.403764	1.576354	1.326500	1.245990	1.407011
N	1.309293	1.232622	1.385964	1.308350	1.257149	1.359551
P	1.574495	1.263537	1.885453	2.737200	1.841049	3.633350
R	1.778426	1.437405	2.119447	1.563453	1.311219	1.815687
U	0.948165	0.861630	1.034699	1.073029	1.027001	1.119057
S	0.918622	0.455971	1.381273	1.295270	1.100198	1.490343

SI Text 7: Sensitivity to initialization network

To understand the model's sensitivity to the chosen initialization network we initialize it with ten random networks with substantially different characteristics compared to the empirical networks. In particular, we choose Erdös-Renyi random networks that have the same number of nodes as the initialization presented in the main text, $N = 18,805$, where two nodes are linked with the uniform probability of $p = 8.0310^{-5}$, such that the expected number of links, $\langle L \rangle$, matches the number of links in the initialization network presented in the main text, $\langle L \rangle = L = 28,391$. In Fig. S7 we show the counter cumulative degree distributions (CCDFs) of all ten initialization networks in green. The sectoral distribution of the firms is the same as in the initialization network.

Except for inititializing Erdös-Renyi networks all other parameters are kept the same as in the main text. In Fig. S7 we show the resulting CCDFs after 500 model iterations in orange. The distributions are all very similar to each other and to the empirical distribution, shown in blue. The average number of nodes in the ten final networks is $\langle N \rangle = 18796$ (std. dev. $\sigma = 135$) and the average number of links is $\langle L \rangle = 28619$ ($\sigma = 701$). Note that these values are ensemble averages over ten model runs, not averages over the iterations of one single model run.

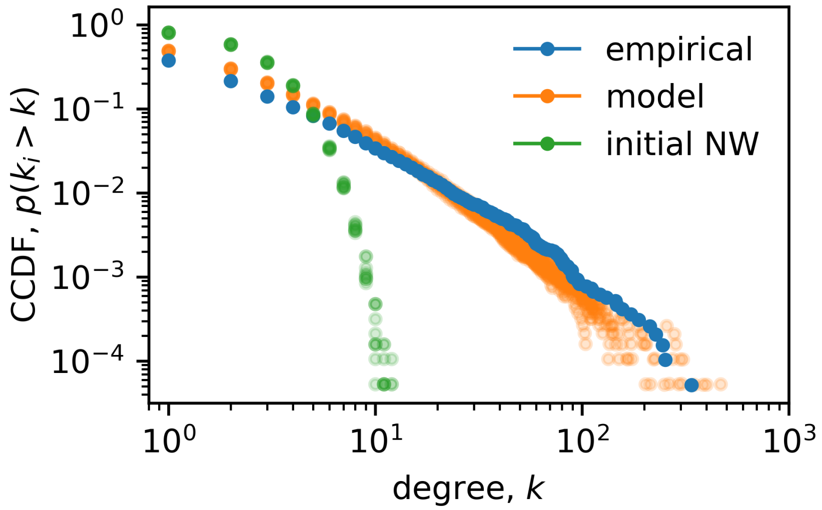

SI Fig. S7. Counter cumulative degree distribution, $p(k_i \geq k)$ for snapshots generated by the supply network generating model (orange) initialized with Erdös-Renyi random networks ($N = 18804$ and $p = 8.0310^{-5}$, green), compared and the empirical degree distribution in January 2017 (blue). The modeled degree distributions are very similar to the empirical. We show 10 different initializations, evolved for 500 iterations.)

The results are also robust to less dramatic changes in the initialization network, such as initializing with the configuration model of the empirical network, or randomizing the sector affiliations of the initial network; not shown here.

SI Text 8: Characterization of nodes that enter

When nodes enter, they are assigned a combination of in- and outdegree, $(k^{in,0}, k^{out,0})$, sampled from the empirical distribution of in- and outdegrees at node entry. In Fig. S8a we show the empirical probability distribution of indegrees at node entry, $p(k^{in,0})$. Most of the weight is concentrated on $k^{in,0} < 2$, with the most common value at 0. Figure S8b shows the empirical probability distribution of outdegrees at node entry, $p(k^{out,0})$. Again, most of the weight is concentrated on $k^{out,0} < 2$, however, with the most common value at 1. In the network generative model, we use the joint distribution, $p(k^{in,0}, k^{out,0})$, shown in Fig. S8c. Notably, not all values occur, and the entry events with highest k^{in} and k^{out}, occur with no out- or in-links, respectively. Most importantly, however, is the underrepresentation of the degree combination $(k^{in,0}, k^{out,0}) = (1, 1)$, as can be seen by comparing the empirical distribution in panel Fig. S8c with the joint probability distribution calculated from the marginals under the assumption of independence, $p'(k^{in,0}, k^{out,0}) = p(k^{in,0})p(k^{out,0})$, shown in Fig. S8d. For our modelling exercise we truncate the empirical distribution at $k \leq 3$, covering 99.8% of the probability weight.

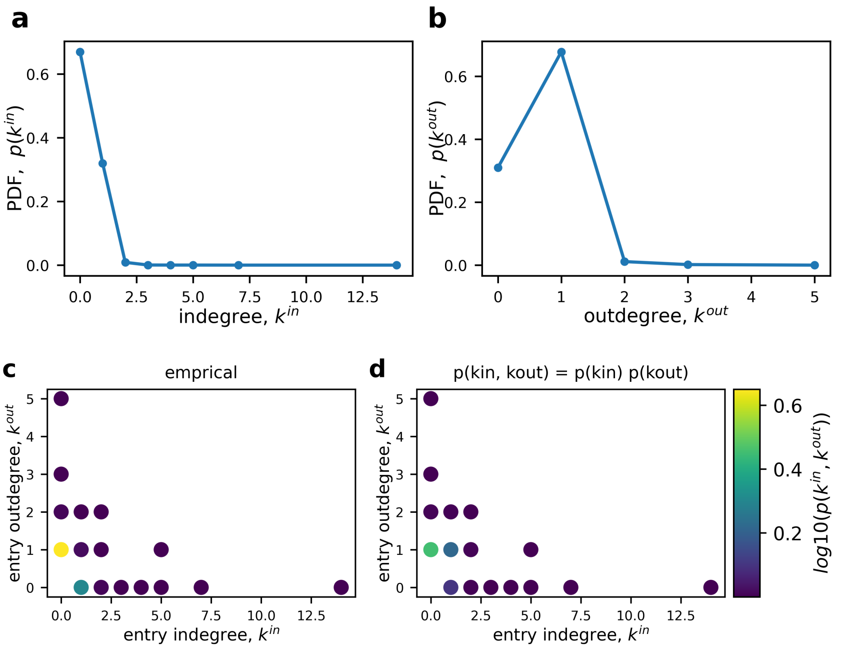

SI Fig. S8. Degree upon entry. (a) Empirical marginal probability distribution of indegrees at node entry, $p(k^{in,0})$, and (b) empirical marginal probability distribution of outdegrees at node entry, $p(k^{out,0})$. (c) Empirical joint probability distribution of entry degrees, $p(k^{in,0}, k^{out,0})$. (d) Joint probability distribution calculated as product of the marginal distributions, $p'(k^{in,0}, k^{out,0}) = p(k^{in,0})p(k^{out,0})$. The combination the degree combination $(k^{in,0}, k^{out,0}) = (1, 1)$ is empirically underrepresented.

SI Text 9: Additional model results

Additional results period A:
In this SI Text we provide additional model results for the year 2017, period A. In Fig. S9a we show the number of nodes, $N(t)$, for every model timestep t. The model slightly underestimates the empirical number of nodes, N_0 (horizontal line). Figure S9b shows the number of links, $L(t)$, for every model timestep t. The model fluctuates around the empirical number of links, L_0 (horizontal line).

Figure S10a compares the empirical indegree distribution (blue) with the indegree distribution of ten model snapshots (vertical lines in Fig. S9. The distributions match well, with a slight underestimation for high k^{in}. Figure S10b compares the empirical outdegree distribution (blue) with the outdegree distribution of ten model snapshots (vertical lines in Fig. S9. The distributions match well across the whole range of k^{out} values.

In Fig. S11 we plot the monthly changes in N and L. Both quantities are negatively skewed with long negative tail. The skewness is caused by cascades of nodes exits, where a large node is removed and many of its neighbors become isolated and, hence, removed from the network.

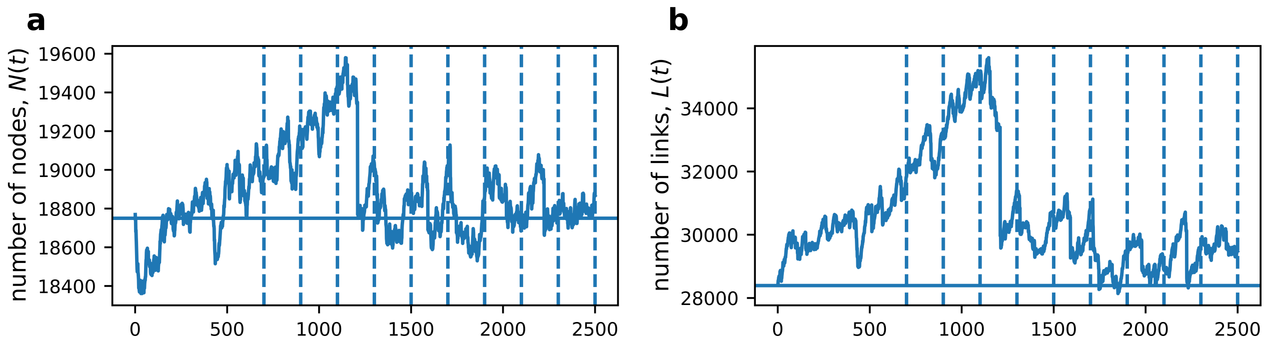

SI Fig. S9. Evolution of the network size as function of model timestep. (a) Number of nodes, $N(t)$, as function of model time t. The horizontal line shows the average number of nodes in the empirical PN, the dashed vertical line marks the networks that were used as snapshots to study the network characteristics. (b) Number of links, $L(t)$, as function of model time t. The horizontal line shows the average number of links in the empirical PN, the dashed vertical lines mark the same snapshots as in (a).

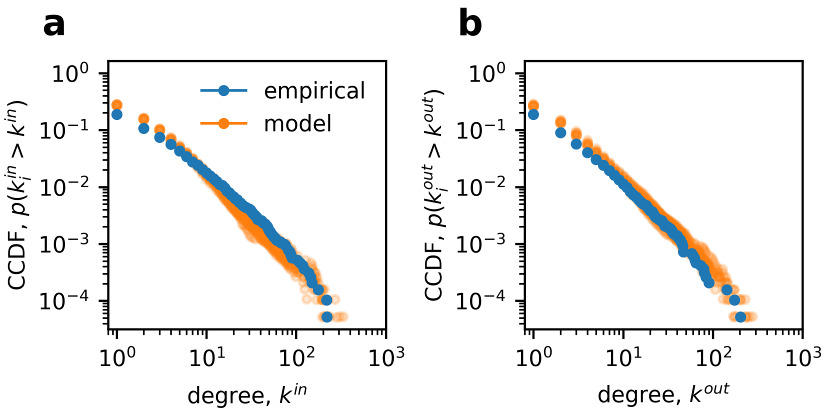

SI Fig. S10. Modelled in- and outdegree for 2017. (a) Counter cumulative distribution for k^{in}, $p(k_i^{in} > k^{in})$. The empirical distribution for Jan. 2017 is shown in blue, the distribution of ten model snapshots in orange. (b) Counter cumulative distribution for k^{out}, $p(k_i^{out} > k^{out})$. The empirical distribution for Jan. 2017 is shown in blue, the distribution of ten model snapshots in orange.

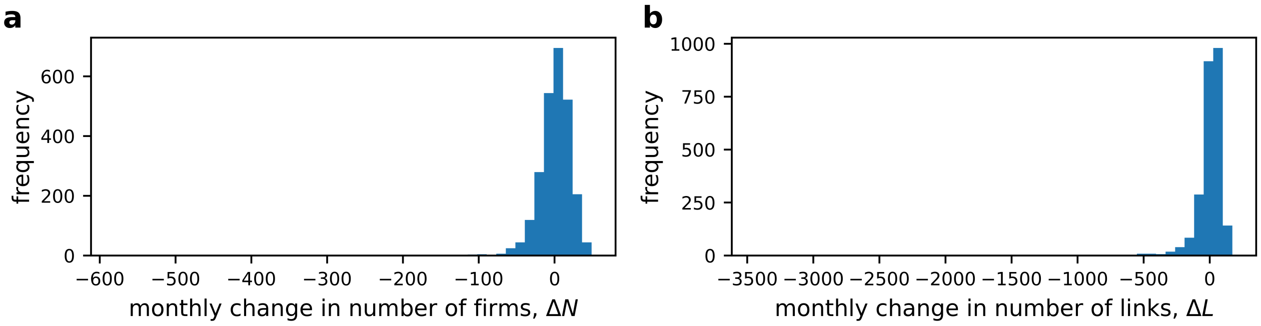

SI Fig. S11. Distribution of monthly change in modeled network size variables in the model run shown in Fig. S9 (period A). (a) Month-on-month difference in the number of firms, ΔN, and (b) the number of links, ΔL. Both quantities are heavily negatively skewed, with negative outliers caused by cascades of node exits.

Additional results period B:
In this SI Text we provide the model results for period B, produced by the parameters specified in Tab. **??**.

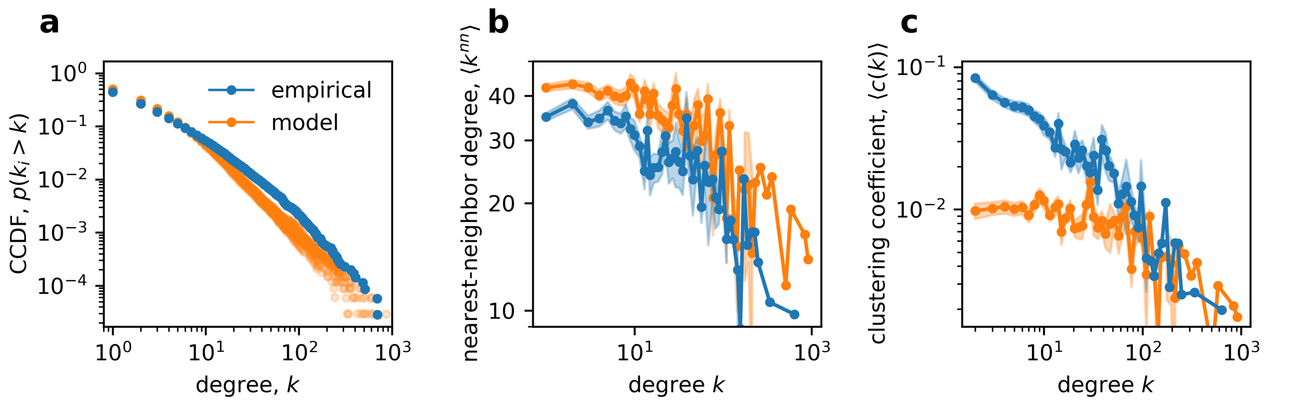

SI Fig. S12. Model results 2019. (a) Counter cumulative degree distribution, $p(k_i \geq k)$ for snapshots generated by the supply network generating model (orange) and the empirical degree distribution in January 2019 (blue). The distributions are very similar. (b) Average nearest neighbor degree for the empirical (blue) and synthetic (orange) network calculated using linear degree bins for $k \leq 10$ and logarithmic degree bins for $k > 10$, the shaded area denotes the standard error. Both networks are disassortative, however, the modeled nearest neighbor degree is higher than the empirical for all degree buckets. (c) Average local clustering coefficient for the eimpirical (blue) and synthetic (orange) network calculated using linear degree bins for $k \leq 10$ and logarithmic degree bins for $k > 10$, the shaded area denotes the standard error. The local clustering coefficient is well captured for large degrees, but underestimated by up to 85% for low degrees.

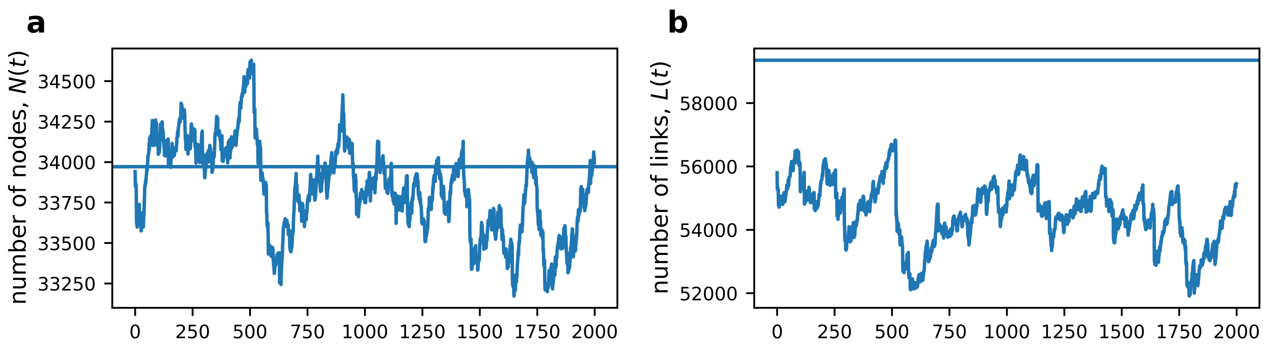

SI Fig. S13. Evolution of the network size as function of model timestep for 2019. (a) Number of nodes, $N(t)$, as function of model time t. The horizontal line shows the average number of nodes in the empirical PN, the dashed vertical line marks the networks that were used as snapshots to study the network characteristics. (b) Number of links, $L(t)$, as function of model time t. The horizontal line shows the average number of links in the empirical PN, the dashed vertical lines mark the same snapshots as in (a). Both quantities slightly underestimate the empirical network size.

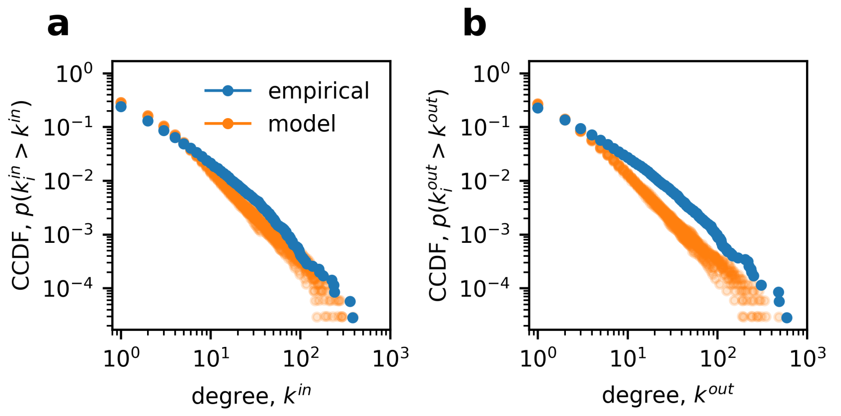

SI Fig. S14. Modeled in- and outdegree for 2019. (a) Counter cumulative distribution for k^{in}, $p(k_i^{in} > k^{in})$. The empirical distribution for Jan. 2019 is shown in blue, the distribution of ten model snapshots in orange. (b) Counter cumulative distribution for k^{out}, $p(k_i^{out} > k^{out})$. The empirical distribution for Jan. 2019 is shown in blue, the distribution of ten model snapshots in orange.

Additional results period C:
In this SI Text we provide the model results for period C, produced by the parameters specified in Tab. **??**.

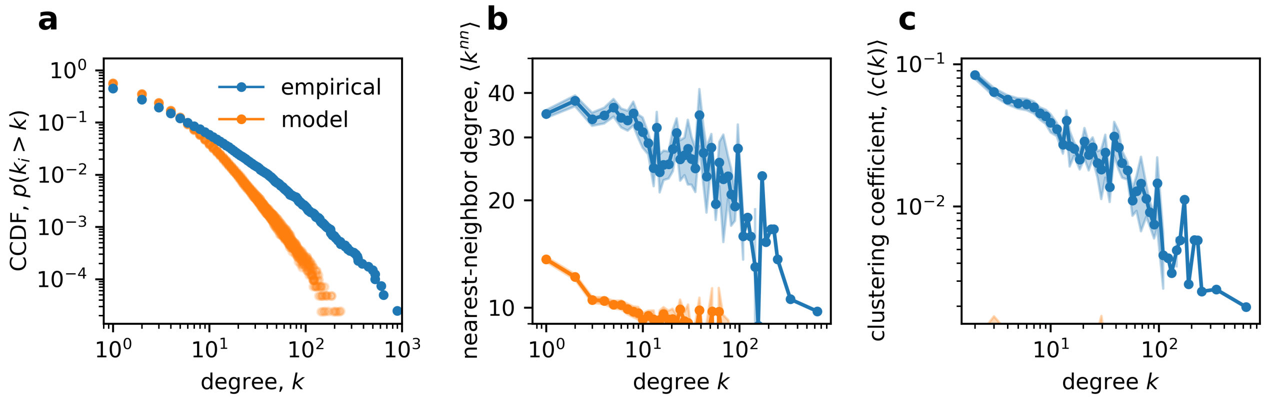

SI Fig. S15. Model results 2021. (a) Counter cumulative degree distribution, $p(k_i \geq k)$ for snapshots generated by the supply network generating model (orange) and the empirical degree distribution in January 2021 (blue). The distributions are very similar. (b) Average nearest neighbor degree for the empirical (blue) and synthetic (orange) network calculated using linear degree bins for $k \leq 10$ and logarithmic degree bins for $k > 10$, the shaded area denotes the standard error. Both networks are disassortative, however, the modeled nearest neighbor degree is higher than the empirical for all degree buckets. (c) Average local clustering coefficient for the eimpirical (blue) and synthetic (orange) network calculated using linear degree bins for $k \leq 10$ and logarithmic degree bins for $k > 10$, the shaded area denotes the standard error. The local clustering coefficient is well captured for large degrees, but underestimated by up to 85% for low degrees.

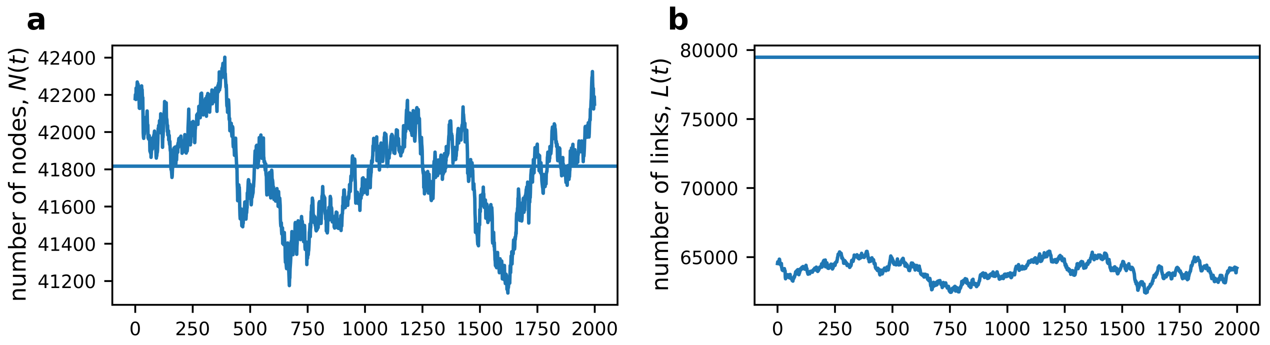

SI Fig. S16. Evolution of the network size as function of model timestep for 2021. (a) Number of nodes, $N(t)$, as function of model time t. The horizontal line shows the average number of nodes in the empirical PN, the dashed vertical line marks the networks that were used as snapshots to study the network characteristics. (b) Number of links, $L(t)$, as function of model time t. The horizontal line shows the average number of links in the empirical PN, the dashed vertical lines mark the same snapshots as in (a). Both quantities slightly underestimate the empirical network size.

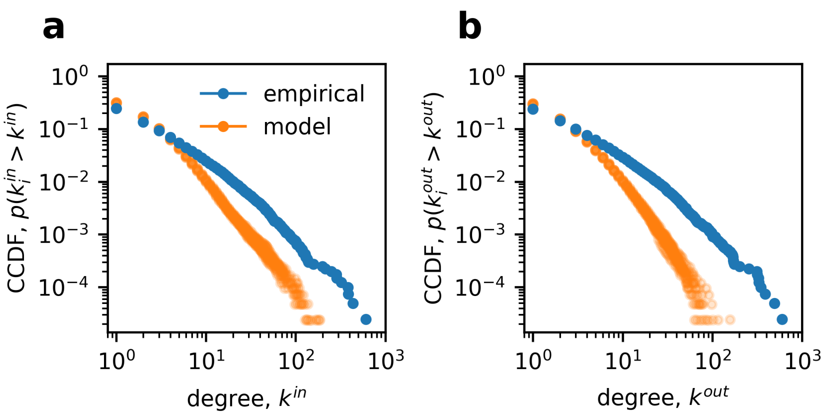

SI Fig. S17. Modeled in- and outdegree for 2021. (a) Counter cumulative distribution for k^{in}, $p(k_i^{in} > k^{in})$. The empirical distribution for Jan. 2021 is shown in blue, the distribution of ten model snapshots in orange. (b) Counter cumulative distribution for k^{out}, $p(k_i^{out} > k^{out})$. The empirical distribution for Jan. 2021 is shown in blue, the distribution of ten model snapshots in orange.

SI Text 10: Link filtering and the high systemic risk core

The empirical ESRI profile presented in Fig. **??** is calculated using different specifications of the algorithm than in the original publication [7].

In particular, the original algorithm accounts for the fact that final demand and exports are not observed in the VAT data. As a consequence, the observed out-strength, s_j^{out}, of firm j in the VAT network is smaller than its total revenue, r_j. This implies that disruptions originating from downstream business customers can affect only a fraction s_j^{out}/r_j of firm j's output. To account for this, the algorithm scales the matrix elements tracking the impact of upstream disruptions from firm i to firm j, Λ_{ji}^{u}, by the factor s_j^{out}/r_j, thereby limiting the maximal impact a disruption to i can have on j. Analogously, the algorithm corrects for the fact that not all of a firm's (material) costs, c_j, are captured in the VAT data, for example due to imports. In this case, the downstream impact Λ_{ji}^{d} is scaled by s_j^{in}/c_j, where s_j^{in} denotes the in-strength of firm j.

In the original publication [7], the aggregate correction factors are $(\sum_{i=1}^{n} s_i^{out})/(\sum_{i=1}^{n} r_i(0)) = 0.60$ and $(\sum_{i=1}^{n} s_i^{in})/(\sum_{i=1}^{n} c_i(0)) = 0.76$. In our case, monthly revenue and cost data are not available, and we therefore set both correction factors to one. As a result, we expect the impact of firm failures—and hence ESRI—to be overestimated.

Further, we don't consider weights, because they are not modeled in the network generative model.

Finally, the data used in [7] aggregates all transactions in a full year (with transactions in at least two distinct quarters), whereas we consider monthly data and a more restrictive filtering procedure.

These changes result in the absence of a characteristic feature of ESRI, the formation of a *high systemic risk core* of firms that all have a similar and high ESRI, visible as a plateau in the rank ordered distribution (the ESRI "profile") [7, 13]. In Fig. S18 we analyze the effects of changing the specifications on the ESRI profile.

In Fig. S18a we plot ESRI as calculated in [7] (blue) and ESRI calculated without revenue correction (orange). Firms are exposed to shocks in the PN much more strongly, raising the ESRI for the firms in the plateau from ca. 0.2 to ca. 0.4. The number of firms in the high systemic risk core, however, stays approximately the same.

We compare ESRI calculated on the weighted (blue) and unweighted (orange) network of the first half year of 2017 in Fig. S18b. The average ESRI, also for firms in the ESRI-plateau, is lowered dramatically, and the plateau contains fewer firms. Omitting link weights reduces the market share of many companies, resulting in a higher replaceability factor and lower ESRI.

Finally, in Fig. S18c we reduce the time window from the first half year in 2017 (blue), to January 2017 (orange) and January 2017 in the network with only stable links, i.e. links with at least three transactions in a six month window (green). The magnitude of ESRI does not change much, but the plateau gets shorter and vanishes for the filtered network. In every step we exclude more links, causing the systemic risk core to become disconnected.

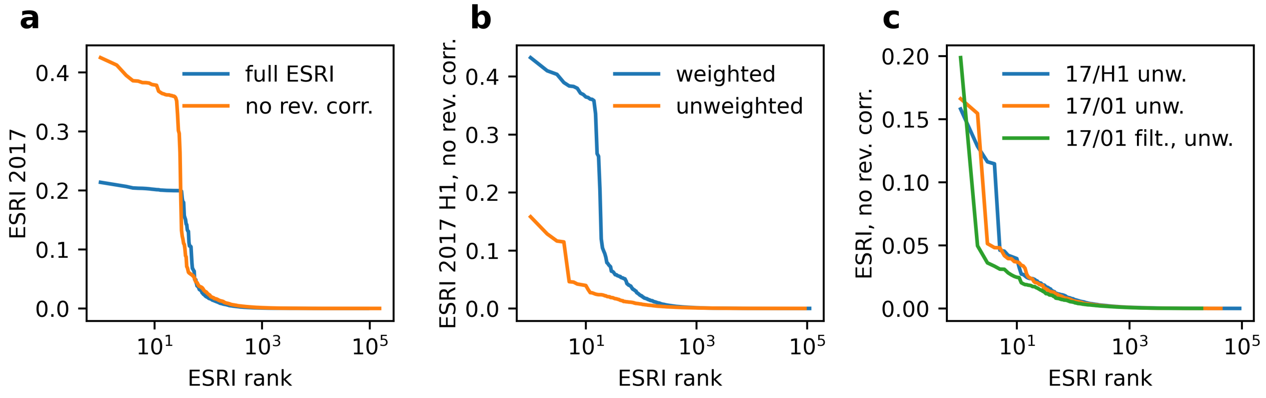

SI Fig. S18. ESRI profile for different parametrizations and time periods. (a) Full ESRI profile for 2017 as in [7] (blue) and without revenue correction (orange). (b) ESRI for the first half year of 2017 without revenue correction for the weighted (blue) and unweighted (orange) network. (c) ESRI without revenue correction and without weights for the first half year of 2017 (blue), for Jan. 2017 (orange) and for Jan. 2017 in the filtered network where links are only contained if they occur three times in a six month window (green).

SI Text 11: Details on Hungarian VAT reporting

Hungarian VAT rates range from a 27% base rate to a 18% and 5% reduced tax rate for certain foods, pharmaceuticals, etc., and there is a 0% rate for public transport [6]. Firms that had a net VAT remittance payments lower than 250,000 HUF in the year before the previous and less than 50 million HUF revenue without taxes, can report their suppliers on an annual basis. Firms exceeding these thresholds but that are below a net VAT remittance of 1 million HUF report their suppliers on a quarterly basis, the remaining firms report their transactions monthly. Firms that exceed one of these thresholds within a given year have to change to the reporting frequency that applies.

SI Text 12: Filtering procedure

To not be sensitive to one-off transactions or missed transactions in otherwise stable links, we filter for only stable links. In Fig. S19 we schematically illustrate he filtering procedure. The data consists of transaction data (blue dots) for each month. We define a link as active if it is present at least three times in a six month window (red dashed line). The link enters (s^+) at the first time step where the condition is fulfilled and exits (s^-) at the first time after the condition is fulfilled (black vertical lines). The filtering procedure is implemented using `R`'s `filter` function.

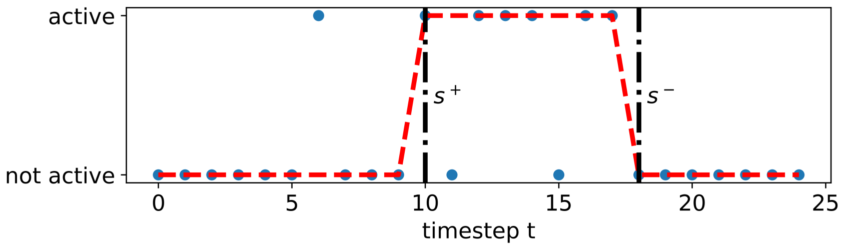

SI Fig. S19. Filtering procedure. Blue dots show in which timestep (month) a transaction occurs. A link is considered active if it is activated at least three times in a six month window (red dashed line). We define link entry (s^+) as the first time step where the condition is fulfilled and exit (s^-) as the first time after the condition is fulfilled (black vertical lines).

The definition we use in our paper is designed to identify recurring economic relationships while preserving the high temporal granularity of the VAT data. The six-month window allows us to focus on sub-annual dynamics, and the requirement that a link is active in at least half of the window filters out sporadic or one-off transactions.

In Fig. S20 we show the remaining transaction volume after filtering with different transaction-window combinations. First, when following an approximate "half-of-the-window" criterion, and requiring transactions in at least 2 out of 3, 3 out of 6, or 6 out of 12 months, the relative volume after filtering remains between 75% and 80%. Second, at least for the 6- and 12-month windows, increasing or decreasing the minimal number of required transactions by one changes the retained volume by less than 10% and 5%, respectively. Although these filtering procedures clearly have an impact on the observed network, their effects are robust to slight variations in the chosen parameters.

We do not condition link stability on transaction size, as our objective is to identify persistent supply-chain relationships rather than infrequent but potentially large transactions associated with investment or one-off procurement.

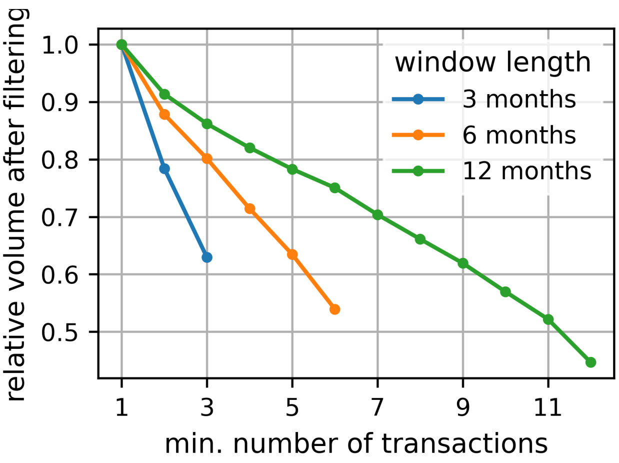

SI Fig. S20. Share of total transaction volume retained under alternative definitions of link stability using different window lengths and proportional minimum-transaction thresholds.

References

1. Enghin Atalay, Ali Hortacsu, James Roberts, and Chad Syverson. Network structure of production. *Proceedings of the National Academy of Sciences*, 108(13):5199–5202, 2011.
2. Pierre-Alexandre Balland. Proximity and the evolution of collaboration networks: evidence from research and development projects within the global navigation satellite system (gnss) industry. *Regional studies*, 46(6):741–756, 2012.
3. Pierre-Alexandre Balland, Mathijs De Vaan, and Ron Boschma. The dynamics of interfirm networks along the industry life cycle: The case of the global video game industry, 1987–2007. *Journal of Economic Geography*, 13(5):741–765, 2013.
4. Vasco M Carvalho and Nico Voigtländer. Input diffusion and the evolution of production networks. Technical report, National Bureau of Economic Research, 2014.
5. Thomas Chaney. The Network Structure of International Trade. *American Economic Review*, 104(11):3600–3634, November 2014.
6. Deloitte. Taxation and Investment in Hungary (rates are updated to 2017) (PDF), 2015. https://www2.deloitte.com/content/dam/Deloitte/ global/Documents/Tax/dttl-tax-hungaryguide-2015.pdf, retrieved 30th august 2021.
7. Christian Diem, András Borsos, Tobias Reisch, János Kertész, and Stefan Thurner. Quantifying firm-level economic systemic risk from nation-wide supply networks. *Scientific reports*, 12(1):7719, 2022.
8. Stanislao Gualdi and Antoine Mandel. On the emergence of scale-free production networks. *Journal of Economic Dynamics and Control*, 73:61–77, 2016.
9. Wataru Miura, Hideki Takayasu, and Misako Takayasu. Effect of Coagulation of Nodes in an Evolving Complex Network. *Physical Review Letters*, 108(16):168701, April 2012.
10. Philipp Mundt. The formation of input–output architecture: Evidence from the european union. *Journal of Economic Behavior & Organization*, 183:89–104, 2021.
11. Ezra Oberfield. A theory of input–output architecture. *Econometrica*, 86(2):559–589, 2018.
12. Jun'ichi Ozaki, Eduardo Viegas, Hideki Takayasu, and Misako Takayasu. Integration of B-to-B trade network models of structural evolution and monetary flows reproducing all major empirical laws. *Scientific Reports*, 14(1):4628, February 2024. Publisher: Nature Publishing Group.
13. Tobias Reisch, Georg Heiler, Christian Diem, Peter Klimek, and Stefan Thurner. Monitoring supply networks from mobile phone data for estimating the systemic risk of an economy. *Scientific reports*, 12(1):13347, 2022.
14. Serguei Saavedra, Felix Reed-Tsochas, and Brian Uzzi. Asymmetric disassembly and robustness in declining networks. *Proceedings of the National Academy of Sciences*, 105(43):16466–16471, 2008.
15. Koutarou Tamura, Hideki Takayasu, and Misako Takayasu. Diffusion-localization transition caused by nonlinear transport on complex networks. *Scientific Reports*, 8(1):5517, April 2018.
